# A Stretch of Negatively Charged Amino Acids of Linker for Activation of T-Cell Adaptor Has a Dual Role in T-Cell Antigen Receptor Intracellular Signaling

**DOI:** 10.3389/fimmu.2018.00115

**Published:** 2018-02-02

**Authors:** Mikel M. Arbulo-Echevarria, Isaac Narbona-Sánchez, Cecilia M. Fernandez-Ponce, Inmaculada Vico-Barranco, Mª Dolores Rueda-Ygueravide, Michael L. Dustin, Arkadiusz Miazek, Mª Carmen Duran-Ruiz, Francisco García-Cózar, Enrique Aguado

**Affiliations:** ^1^Department of Biomedicine, Biotechnology and Public Health (Immunology), Core Research Facility for Health Sciences, University of Cádiz and Puerto Real University Hospital Research Unit, Cádiz, Spain; ^2^Servicio de Neumología-Alergia, Hospital Puerta del Mar, Cádiz, Spain; ^3^Nuffield Department of Orthopaedics, Rheumatology and Musculoskeletal Sciences, The Kennedy Institute of Rheumatology, The University of Oxford, Headington, United Kingdom; ^4^Hirszfeld Institute of Immunology and Experimental Therapy, Polish Academy of Sciences, Wroclaw, Poland; ^5^Department of Biomedicine, Biotechnology and Public Health (Biochemistry), University of Cádiz, Cádiz, Spain; ^6^Institute of Biomedical Research Cadiz (INIBICA), Cádiz, Spain

**Keywords:** LAT, TCR, CD3, signaling, Lck

## Abstract

The adaptor protein linker for activation of T cells (LAT) has an essential role transducing activatory intracellular signals coming from the TCR/CD3 complex. Previous reports have shown that upon T-cell activation, LAT interacts with the tyrosine kinase Lck, leading to the inhibition of its kinase activity. LAT–Lck interaction seemed to depend on a stretch of negatively charged amino acids in LAT. Here, we have substituted this segment of LAT between amino acids 113 and 126 with a non-charged segment and expressed the mutant LAT (LAT-NIL) in J.CaM2 cells in order to analyze TCR signaling. Substitution of this segment in LAT prevented the activation-induced interaction with Lck. Moreover, cells expressing this mutant form of LAT showed a statistically significant increase of proximal intracellular signals such as phosphorylation of LAT in tyrosine residues 171 and 191, and also enhanced ZAP70 phosphorylation approaching borderline statistical significance (*p* = 0.051). Nevertheless, downstream signals such as Ca^2+^ influx or MAPK pathways were partially inhibited. Overall, our data reveal that LAT–Lck interaction constitutes a key element regulating proximal intracellular signals coming from the TCR/CD3 complex.

## Introduction

T lymphocytes are activated upon the recognition of peptides bound to MHC molecules in the surface of antigen-presenting cells (APCs). Antigen recognition by T cells is performed by the T-cell antigen receptor (TCR) heterodimer, triggering intracellular signals leading eventually to proliferation, production of cytokines, and differentiation of T lymphocytes, which are essential for proper immune responses (1, 2). Most of T cells express a TCR consisting of a TCR-αβ heterodimer associated with CD3ε, γ, δ, and ζ polypeptides, constituting the TCR/CD3 complex. The cytoplasmic tails of CD3 polypeptides contain immunoreceptor tyrosine-based activation motifs (ITAMs), which are essential for the transduction of intracellular signaling from the TCR/CD3 complex. Upon TCR recognition of a foreign antigen, the Src family kinase Lck phosphorylates tyrosine residues of CD3 ITAMs, which in turn leads to the recruitment of the cytosolic tyrosine kinase ZAP70. Once there, ZAP70 is phosphorylated and activated by Lck, allowing the phosphorylation of adaptor proteins LAT and SLP-76. Once their tyrosines are phosphorylated, LAT and SLP-76 bind to several cytosolic proteins leading to the formation of multiprotein complexes, denominated signalosomes, which are critical for the amplification and diversification of intracellular downstream signaling pathways leading to activation, proliferation, and differentiation of naive T lymphocytes. The flexibility of the signaling network is thought to account for the ability of T cells to exhibit different physiological responses, each of which requires engagement of the TCR (1, 3).

The membrane adaptor LAT has a critical function in the transduction of intracellular signals coming from the TCR/CD3 complex (4, 5). LAT is an adaptor protein with a short extracellular N-terminal fragment, a hydrophobic transmembrane segment followed by a long cytoplasmic tail containing nine conserved tyrosine residues (6, 7). The essential role of LAT for TCR/CD3 signal transduction was first shown in LAT-deficient T-cell lines, which showed defects in activation-induced intracellular calcium, Ras activation, and IL-2 production, defects that were relieved upon reintroduction of LAT in these cells (8, 9). Moreover, LAT-knockout (LAT-KO) mice revealed its essential role for T-cell development: LAT-KO mice show a total block in thymic maturation at the CD4^−^CD8^−^ double negative (DN) stage and contain no mature T lymphocytes (10). It has also been demonstrated, both *in vitro* and *in vivo*, the critical role of LAT tyrosine residues for the proper transduction of activation signals from the TCR/CD3 complex (11–13). Unexpectedly, a LAT-knockin (LAT-KI) strain of mice in which the sixth tyrosine was mutated to phenylalanine (LAT^Y136F^) exhibited a partial block in T-cell maturation, but they later developed a polyclonal lymphoproliferative disorder of CD4 T cells producing massive amounts of T helper type 2 (TH2) cytokines, which caused tissue eosinophilia and maturation of plasma cells secreting huge amounts of IgE and IgG1 immunoglobulins (14, 15). In the same way, another KI strain of mice in which the last three carboxy-terminal tyrosine residues were mutated to phenylalanines (LAT^3YF^) showed a total block in αβ T-cell development and a partially impaired γδ T-cell development but developed a lymphoproliferative disorder of γδ T cells that chronically produced TH2 cytokines (16). These paradoxical phenotypes established for the first time that LAT is a crucial regulator of T-cell homeostasis, later confirmed in conditional LAT-KI strains of mice (17).

In a search of non-tyrosine-based motifs in LAT sequence explaining its regulatory functions, our group has analyzed the role of proteolytic cleavage as a terminator of TCR signaling (18, 19). Upon proapoptotic stimuli, LAT is cleaved at three aspartic acid residues which are located close to relevant tyrosine residues and, interestingly, LAT phosphorylation inhibits its Fas-mediated cleavage. This cleavage generates a truncated form of LAT unable to transduce intracellular signals, suggesting a mechanism of termination of signals in T cells. Consistently, the related adaptor molecule NTAL (LAB/LAT2) is also subject of proteolytic cleavage after Fas stimulation in B cells and monocytes (20). Interestingly, it has been previously shown that upon T cells’ activation LAT is able to bind and negatively regulate Lck kinase and this interaction is mediated by a negatively charged stretch between amino acids 112 and 126 of LAT (21, 22). Thus, the truncated form of LAT at aspartic 126 would still be able to bind Lck, constituting a putative new mechanism for negative regulation of the TCR/CD3 signaling cassette. Here, we have generated a new mutant of LAT in which the negatively charged stretch has been substituted by a non-charged amino acid sequence and expressed this mutant form of LAT in J.CaM2 cells. Substitution of this negatively charged segment of LAT prevented the activation-induced interaction with Lck, and we called this mutant LAT-NIL (from Not Interacting with Lck). Furthermore, expression of LAT-NIL increased several early intracellular signals, including phosphorylation of ZAP70 and LAT Tyr residues 171 and 191, although downstream signals such as Ca^2+^ influx and MAPK pathways were partially inhibited. Overall, our data suggest that LAT–Lck interaction is crucial for the termination of intracellular signaling triggered upon TCR engagement.

## Materials and Methods

### Antibodies and Reagents

The anti-Fas (IgM) antibody, anti-phospho-LAT-Tyr226, and rabbit polyclonal anti-human LAT were from Merck-Millipore; anti-PLC-γ1 mAb, anti-Lck, and anti-Erk were from Santa Cruz Biotechnology (Santa Cruz, CA, USA); antibodies binding phospho-Erk, phospho-PLC-γ1-Tyr783, phospho-ZAP70-Tyr319, phospho-MEK-Ser221, phospho-LAT-Tyr191, and anti-MEK were from Cell Signaling Technology; anti-6His-HRP, anti-Grb2, and anti-phospho-LAT-Tyr132 antibodies were from Abcam (Cambridge, MA, USA); and anti-CD69-APC (allophycocyanin) and anti-β-actin monoclonal antibodies were from Biolegend. The protein synthesis inhibitor cicloheximide was purchased from Merck-Millipore. Stimulations were performed with anti-human CD3 (OKT3; eBioscience) or anti-human CD28 (CD28.2; BD Pharmingen) monoclonal antibodies.

### Cell Culture

The LAT-deficient J.CaM2 cell line was generously provided by Dr. Arthur Weiss, University of California, San Francisco (CA, USA). Cells were grown in complete RPMI 1640 medium (Lonza) supplemented with 10% FCS (Lonza) and 2 mM l-glutamine at 37°C in a humidified atmosphere containing 10% CO_2_.

### Mutagenesis and Lentiviral Transduction

LAT cDNA cloning was performed as previously described (18). Site-directed mutagenesis was performed to change the sequence for segment between residues 112 and 126. Coding sequences in the plasmids were verified by sequencing and then subcloned in frame with GFP in the SIN lentiviral transfer plasmid pHR’SINcPPT-Blast by means of site-specific recombination (Gateway LR Clonase; Invitrogen). Lentiviral supernatants were generated as previously described (18) and used to induce expression of wild-type (WT)-LAT or the LAT-NIL mutant in J.CaM2 cells. Blasticidin selection (20 µg/ml) was applied to transduced cells after 72 h of culture, and expression of GFP was analyzed by means of FACS analysis (CytoFLEX; Beckman Coulter).

### Preparation of Cell Lysates and Western Blotting

Lentivirally transduced J.CaM2 cells were starved in RPMI 1640 without FCS for 18 h before being stimulated with anti-CD3 mAb at 37°C. Cells were then lysed at 2.5 × 10^7^ cells/ml in 2× Laemmli buffer, followed by incubation at 99°C for 5 min and sonication. For anti-Fas stimulation, cells were incubated with 100 ng/ml of anti-Fas mAb at 1 × 10^6^ cells/ml in RPMI 1640, supplemented with 10% FCS, and then pelleted and lysed as described earlier. For Western blotting, whole-cell lysates were separated by SDS-PAGE and transferred to PVDF membranes, which were incubated with the indicated primary antibodies, followed by the appropriate secondary antibody conjugated to IRDye 800CW (Li-Cor, Lincoln, NE, USA) or horseradish peroxidase (HRP). Reactive proteins were visualized using the Odyssey CLx Infrared Imaging System (Li-Cor) or by enhanced chemiluminescence (ECL) acquired in a ChemiDoc Touch Imaging System (Bio-Rad Laboratories). For reprobing, PVDF membranes were incubated for 10 min at room temperature with WB Stripping Solution (Nacalai Tesque, Kyoto, Japan), followed by a TTBS wash. For the cycloheximide chase assay, cells were treated with 0.1 mM cycloheximide for up to 10 h. Every 2 h, cell samples were lysed in 2× Laemmli buffer and LAT protein levels were determined by immunoblotting and quantified by densitometry.

### Immunoprecipitations

For LAT immunoprecipitation, J.CaM2 cells expressing T-LAT or LAT-NIL were stimulated with pervanadate (0.01 mM sodium vanadate/4.5 mM H_2_O_2_, freshly prepared), PHA (20 µg/ml) or anti-CD3 plus anti-CD28 antibodies were conducted at 37°C for 5 min, and subsequently lysed with a buffer containing 20 mM Tris-HCl pH 7.4, 1% Triton X-100, 150 mM NaCl, 1 mM Na_3_VO_4_, 1 mM NaF, and 2 mg/ml each small protease inhibitors. Lysates were incubated on ice with 2 µg of anti-LAT antibody for 45 min; subsequently, lysates were incubated with 20 µl of Bio-Adembeads protein AG-coupled magnetic beads for additional 45 min, washed three times with 500 µl of lysis buffer, and eluted by applying 30 µl of PAG elution buffer (Ademtech, Pessac, France). For Western blotting, immunoprecipitates were separated by SDS-PAGE and transferred to PVDF membranes, which were incubated with the indicated primary antibodies, followed by the appropriate secondary antibody, conjugated to HRP. Reactive proteins were visualized using the ECL system, and images were acquired in a ChemiDoc Touch Imaging System.

### Confocal Microscopy Analysis

Cell imaging of J.CaM2 cells lentivirally transfected with WT-LAT or the LAT-NIL mutant fused to ZsGreen was performed on Olympus BX61WI confocal microscope (Life Science microscopy—Europe). In order to stain cell membranes, cells were labeled with PKH26 red fluorescent cell linker kit (Sigma-Aldrich), according to the manufacturer’s protocol. Cells were collected, washed, resuspended in 500 µl of Diluent C, and mixed with an equal volume of PKH26 ethanolic dye solution diluted in Diluent C to reach a final concentration of 2 µM, and subsequently incubated for 1 minute at RT. The staining was blocked adding 2 ml of fetal bovine serum (FBS). Cells were washed, centrifuged on object slides at 900 rpm for 10 min using Cytospin™ 4 Cytocentrifuge (ThermoFisher Scientific), fixed with 4% Paraformaldehyde/PBS during 20 min at RT, washed with PBS, and mounted using Fluoro-Gel (Electron Microscopy Sciences). Microscope slides containing cells expressing WT-LAT or the LAT-NIL mutant fused to ZsGreen and stained with PKH26 were placed onto the confocal microscope stage. Fluorescence was excited and acquired with the Virtual Channel Scan EGFP and Alexa Fluor 546, and using an Olympus PlanApo N 60× immersion objective. Laser power and detector settings were adjusted in order to avoid autofluorescence detection.

### Measurement of Intracellular Ca^2+^ Mobilization

Measurement of intracellular free Ca^2+^ was carried out using Indo-1 AM (acetoxymethyl) (2 µM; Molecular Probes, Invitrogen) as previously described (18). Calcium measurements were performed using a Synergy MX Multi-Mode Reader (Biotek) at 37°C. Cells were excited by light at a wavelength of 340 nm, and the fluorescence emitted at 405 and 485 nm was collected alternately per second. Calcium mobilization was evaluated by the ratio of 405/485 nm fluorescence signal.

### Statistical Analysis

Western blots were densitometrically quantified, and statistics was performed with Microsoft Excel using a two-tailed *t*-test. Levels of significance *p* < 0.05 and <0.005 are presented as * and **, respectively.

## Results

### Lentiviral Transduction of J.CaM2 Cells

**Figure 1:**
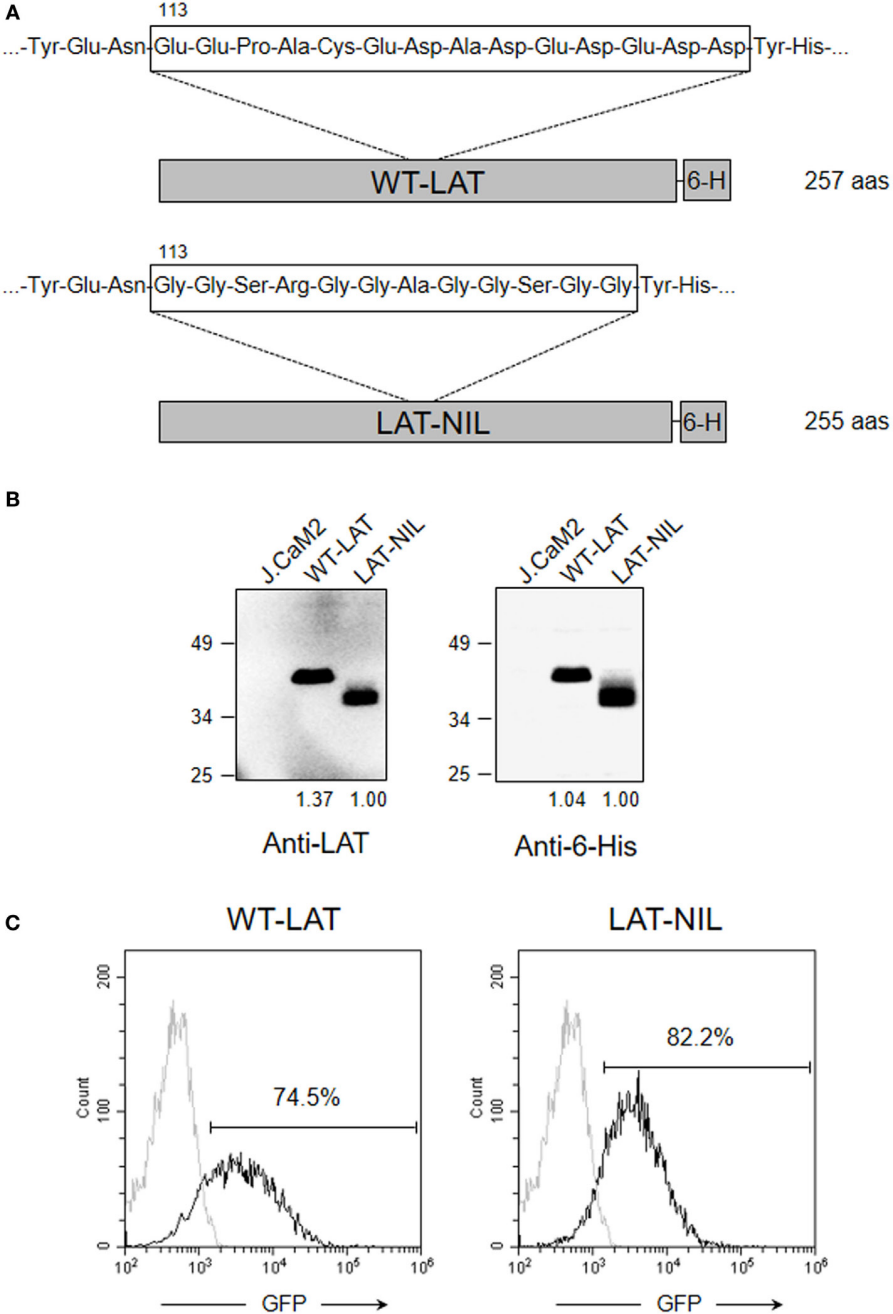
Expression of WT-LAT and LAT-NIL in J.CaM2 cells. **(A)** Schematic representation and amino acid sequence of the negatively charged stretch in WT-LAT or the substituting sequence in LAT-NIL mutant. **(B)** J.CaM2 and lentivirally transduced J.CaM2 cells expressing WT-LAT or LAT-NIL mutant were lysed, and linker for activation of T-cell (LAT) expression was analyzed by Western blot with anti-LAT (left panel) or anti-6His (right panel). Molecular masses in kilodaltons are indicated on the side of the Western blots. Numbers below the blots indicate relative densitometric quantification of the bands. Images are representative of four independent experiments. **(C)** Histograms of GFP expression in J.CaM2 and lentivirally transduced cells expressing WT-LAT or LAT-NIL. Histograms are representative of four independent experiments.

It has been previously shown that activation of T cells induces the interaction between LAT and Lck kinase (21–23). The segment comprising residues 112–126 of human LAT is required for the interaction with Lck, and this sequence is rich in negatively charged amino acids (22). Interestingly, interaction with LAT negatively regulates kinase activity of Lck, suggesting a negative feedback loop of TCR signaling. Concordantly, in LAT-deficient J.CaM2 cells, Lck shows increased kinase activity with regard to Jurkat cells, and restoration of LAT expression decreases its kinase activity (21). At the same time, upon proapoptotic stimuli LAT is cleaved at aspartic residues 126, 138, and 186, leaving an N-terminal fragment that would be able to bind to Lck (18, 22). The amino acid sequence of this segment is evolutionarily conserved, supporting a relevant function (Table 1). This prompted us to analyze the role of this segment of LAT by substituting it with a sequence of amino acids without negative charge (Figure 1A). The original sequence in LAT is 14 amino acids long and has a net charge of -10 at pH 7. We have introduced a segment of 12 amino acids with a net charge of + 1. The introduced sequence incorporates amino acids predicted to have at least the same or higher flexibility than the original sequence, in order to prevent any conformational effect (24). Lentiviral vectors were generated including the coding region of WT-LAT or the LAT-NIL (Not Interacting with Lck) mutant, fused to a 6His tag (Figure 1A). Lentiviral vectors contained an IRES and GFP coding sequence as a reporter of the level of transfection, and J.CaM2 cells lentivirally transduced with those vectors coding for WT-LAT or LAT-NIL mutant expressed similar levels of GFP (Figure 1C). Analysis of cell lysates by Western blot showed similar expression levels for WT-LAT and LAT-NIL mutants, regardless if the Western blot analysis was performed with anti-LAT or anti-6His antibodies (Figure 1B). Interestingly, electrophoretic mobility of LAT-NIL mutant was increased with regard to WT-LAT, although the predicted molecular weight of both fusion proteins was very similar (27.1 kDa for WT-LAT and 26.4 kDa for LAT-NIL).

**Table 1:** Amino acid sequence of the negatively charged fragment of LAT in different species.

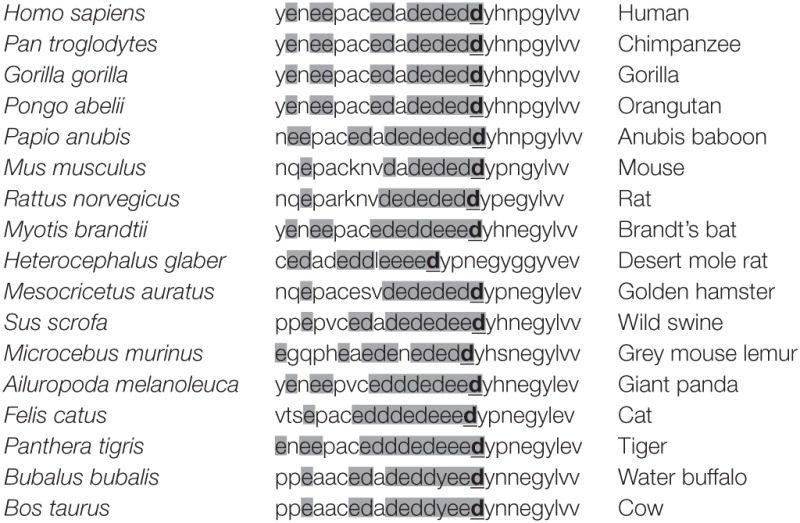

*Amino acid sequence preceding the cleavage point of LAT at Asp 126 in human LAT (bold and underlined) is shown. Shadowed in grey are shown the negatively charged amino acids*.

**Figure 2:**
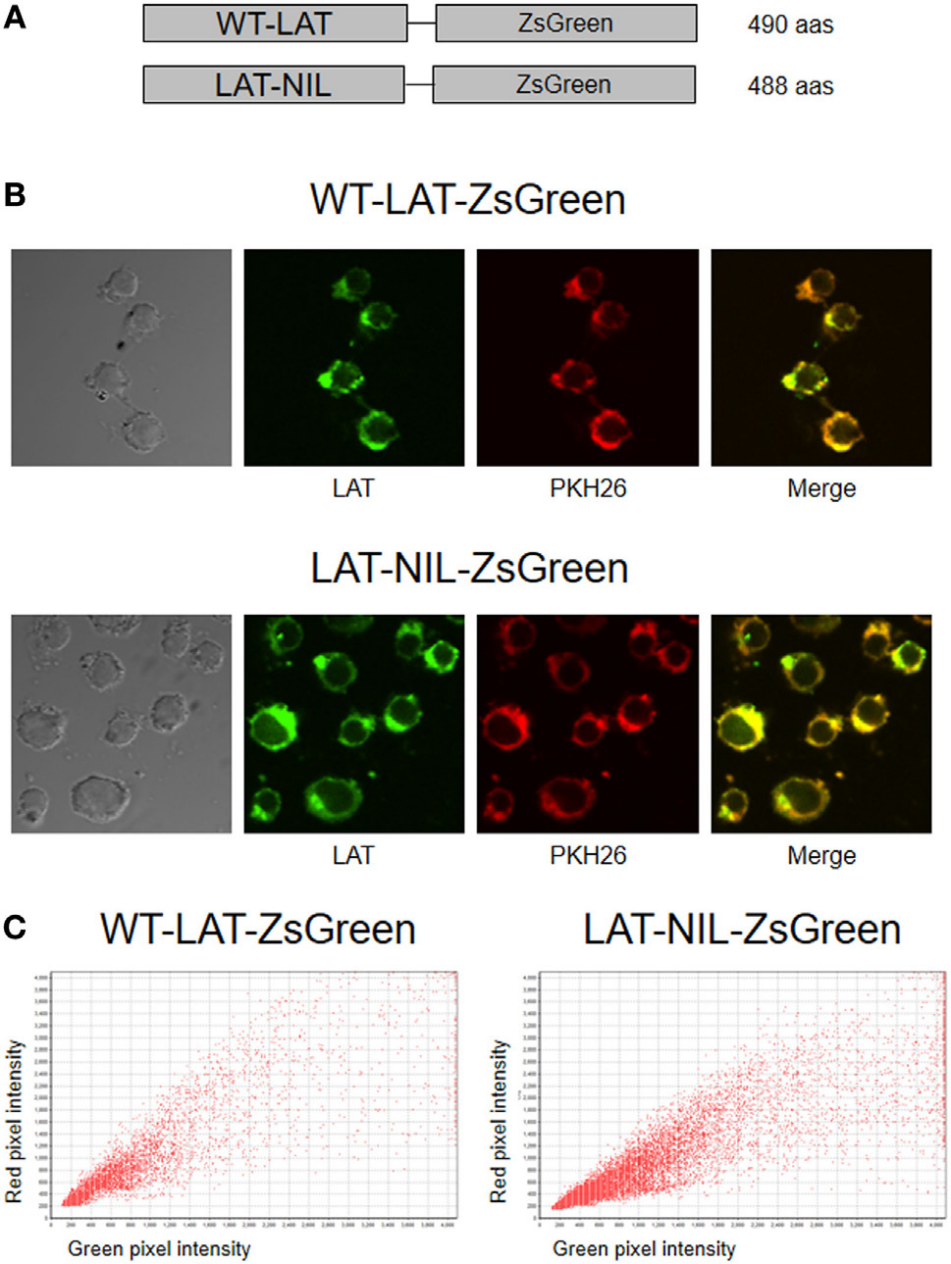
Subcellular localization of LAT-NIL protein. **(A)** Schematic representation of WT-LAT-ZsGreen and LAT-NIL-ZsGreen fusion proteins lentivirally transduced into J.CaM2 cells. **(B)** J.CaM2 cells lentivirally transduced with DNA coding for the fusion proteins of WT-LAT or the LAT-NIL mutant were labeled with 2 µM PKH26 red fluorescent dye, and examined with confocal microscopy. Images were captured at 60× magnification. Images on the right show merged green and red fluorescence. **(C)** Scatterplots of green and red pixel intensities of cells shown in **(B)**.

In order to verify the membrane expression of both types of LAT molecules, lentiviral vectors coding for fusion proteins with ZsGreen were generated (Figure 2A), and J.CaM2 cells were transduced with these vectors. Transduced cells were then labeled with PKH26, a membrane-labeling red fluorescent marker (25), in order to analyze membrane expression of WT-LAT-ZsGreen and LAT-NIL-ZsGreen fusion proteins by confocal microscopy. As it can be seen in Figure 2B, the change introduced in the negatively charged stretch of LAT does not seem to alter the LAT expression pattern and suggests that WT and the LAT-NIL mutant are excluded from the nucleus and distributed in a mix of vesicles and plasma membrane. The analysis of green and red fluorescence in WT-LAT-ZsGreen and LAT-NIL-ZsGreen expressing cells was represented graphically, showing a similar pattern in both types of cells (Figure 2C). Moreover, Pearson’s correlation coefficient (PCC) values of green and red fluorescence were 0.9452 ± 0.0182 for cells expressing WT-LAT-ZsGreen and 0.9066 ± 0.0235 for cells expressing LAT-NIL-ZsGreen. Altogether, our results suggest that the mutation of the negatively charged stretch of LAT does not affect its subcellular localization.

### The Negatively Charged Stretch of LAT Is Needed for Activation-Induced Interaction with Lck

**Figure 3:**
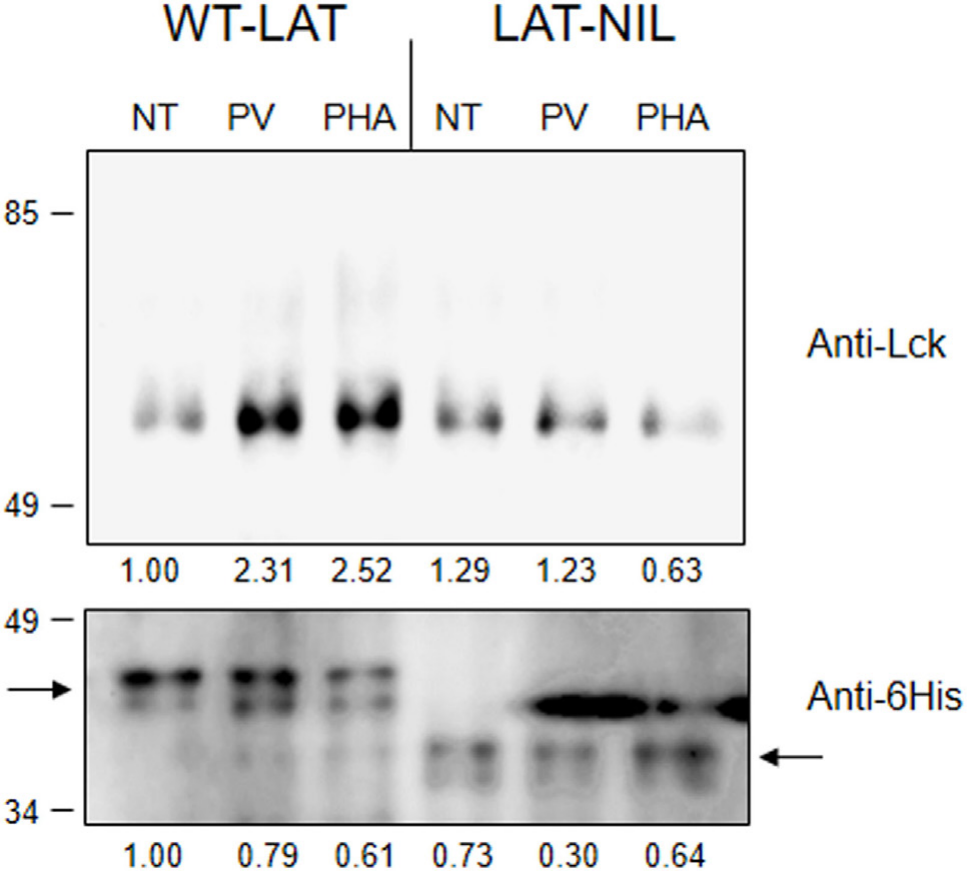
The negatively charged stretch of linker for activation of T cell (LAT) mediates interaction with Lck. 10^7^ J.CaM2 cells expressing either WT-LAT or the LAT-NIL mutant were left untreated (NT) or stimulated for 5 min with 5 µM pervanadate (PV) or 20 µg/ml of PHA, and postnuclear lysates were subjected to LAT immunoprecipitation with an anti-LAT antibody. The precipitates were analyzed by Western blot with anti-Lck antibody (upper panel) or anti-6His antibody (lower panel), as indicated. Images are representative of three independent experiments. Numbers below the blots indicate the densitometric quantification of the corresponding bands.

Next, we sought to verify the role of the negatively charged segment of LAT in the activation-induced interaction with Lck. When it was first cloned, LAT was suggested as a possible molecular partner of Lck, since activated Lck was able to induce low levels of LAT phosphorylation in the absence of ZAP70 (6), and very recently it has been observed that one tyrosine residue of LAT could be phosphorylated by Lck (26). Moreover, an activation-induced interaction of LAT with Lck was previously shown (21, 23, 27) and this interaction appeared to be mediated by a segment between amino acids 113 and 126 in LAT (22). In order to confirm the relevance of this segment of LAT, we proceeded to immunoprecipitate WT-LAT or the LAT-NIL mutant from stimulated J.CaM2 cells expressing either type of LAT isoforms. To achieve a stimulation level able to induce LAT-Lck interaction, we treated cells for 5 min at 37°C with 5 µM pervanadate or 20 µg/ml of PHA. Western blot analysis of the immunoprecipitates showed that cell activation-induced WT-LAT association with Lck, supporting previous results (Figure 3, upper panel). However, neither PHA nor pervanadate was able to increase LAT–Lck interaction in LAT-NIL expressing cells, although the level of immunoprecipitated LAT was similar for both types of cells. Therefore, this result shows that the negatively charged amino acids’ stretch of LAT is needed for the activation-induced interaction with Lck.

### LAT-NIL Mutant Has Reduced Stability

**Figure 4:**
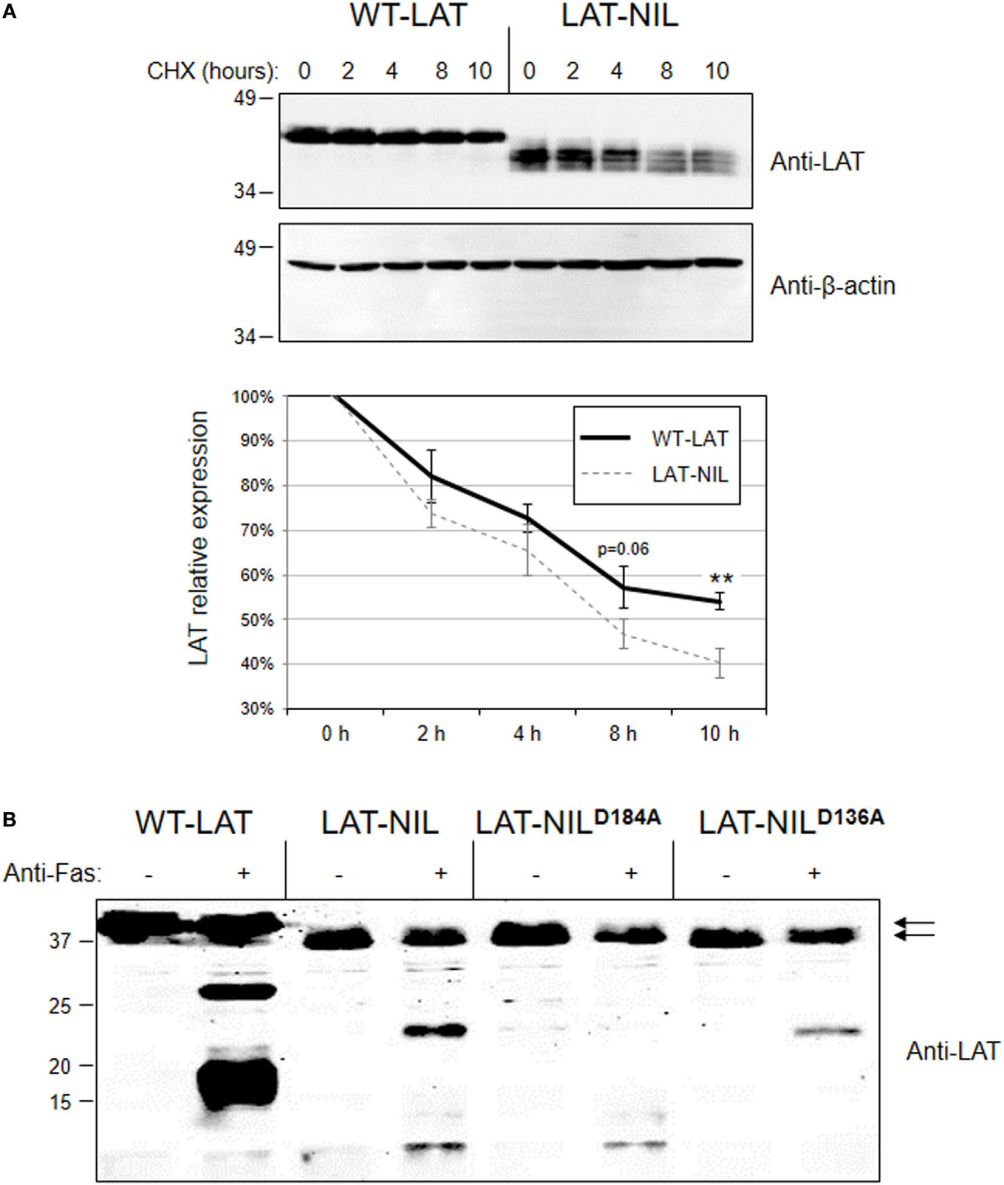
Degradation of WT-linker for activation of T cell (LAT) and LAT-NIL mutant proteins. **(A)** Cell extracts from J.CaM2 cells expressing WT-LAT or the LAT-NIL mutant were treated with cycloheximide (CHX) for indicated time in hours, and LAT (upper panel) or beta-actin (middle panel) protein levels were detected by Western blot. Densitometric analysis was performed over eight experiments and the relative expression of LAT was represented (lower panel). Bars represent the standard error. Asterisks represent statistical significance. **(B)** J.CaM2 cells expressing WT-LAT or the indicated mutants were treated for 4 h at 37°C with 100 ng/ml of anti-Fas antibody, and LAT cleavage was assessed in total cell lysates by Western blot with anti-LAT antibody. Molecular masses in kDa are indicated adjacent to the Western blot. Arrows indicate the bands corresponding to the whole WT-LAT molecule (upper arrow) and LAT-NIL (lower arrow).

We have previously described a functional isoform of LAT originated from an intron 6 retention event generating an in-frame splice variant of LAT mRNA denoted as LAT_i6_ (28). This isoform has been detected at the RNA level in human and other mammalian species, and although the protein product of LATi6 isoform is able to support TCR signaling, it displays a shorter half-life. Interestingly, the amino acid sequence introduced in LAT_i6_ isoform after the intron 6 retention event is located just after a Glu in position 113, at the beginning of the negatively charged stretch of LAT needed for the interaction with Lck. Therefore, we sought to verify if this fragment of LAT is important for the stability of the molecule. Consequently, we measured stability of WT-LAT and LAT-NIL by the cycloheximide chase assay. Lentivirally transduced J.CaM2 cells were incubated in the presence of the translational inhibitor cycloheximide, and cells were collected at specific time points and lysed. Western blot analysis showed that, in agreement to LAT_i6_ isoform, LAT-NIL mutant is degraded with faster kinetics with regard to WT-LAT (Figure 4A, upper panel). Densitometric analysis of eight independent experiments showed that LAT-NIL mutant has a reduced stability with regard to WT-LAT, with statistical significance after 10 h after cycloheximide treatment. Moreover, longer cycloheximide treatments (14 and 18 h) of lentivirally transduced J.CaM2 cells also showed a reduced stability of LAT-NIL mutant (Figure S1A in Supplementary Material). Consequently, these results support a relevant role for this part of LAT sequence in its stability and/or turnover. Given that the negatively charged stretch of LAT is needed for the activation-induced interaction with Lck, we sought to evaluate whether the presence of Lck could modify LAT turnover. Therefore, we performed a cycloheximide chase assay with Jurkat cells and J.CaM1.6 cells deficient in Lck expression. As it can be seen in Figure S1B in Supplementary Material, JCaM1.6 expresses lower levels of LAT with regard to Jurkat cells. Moreover cycloheximide treatment induces a greater loss of LAT expression in J.CaM1.6 cells with regard to Jurkat cells, suggesting that Lck interaction could somehow stabilize LAT.

Next, given that the substituted sequence in LAT-NIL isoform included one of the described cleavage points in LAT (18), we decided to verify if the substitution of this amino acid residue altered the cleavage of LAT induced upon Fas engagement. Therefore, we treated J.CaM2 cells expressing WT-LAT or LAT-NIL with anti-Fas antibody for 4 h at 37°C. As it can be seen in Figure 4B (lanes 1–4), cleavage of LAT-NIL also produced two major proteolytic fragments. The calculated molecular weight of proteolytic fragments generated in WT-LAT upon cleavage at Asp186 and Asp138 are 19.9 and 15.1 kDa, respectively, and the fragments that LAT-NIL should produce upon cleavage of LAT-NIL at Asp184 and Asp136 (LAT-NIL is two amino acids shorter than the WT-isoform) are very similar to the WT counterpart (i.e., 19.2 and 14.4 kDa, respectively). Interestingly, the difference in the electrophoretic mobility of proteolytic fragments generated by LAT-NIL with regard to the ones produced by WT-LAT was greater than expected. Thus, in order to explain the origin of the proteolytic bands observed in LAT-NIL, we generated two additional mutants in which aspartic residues 136 and 184 were mutated to alanine (mutants LAT-NIL^D136A^ and LAT-NIL^D184A^, respectively). Anti-Fas treatment of LAT-NIL^D136A^ expressing cells generated only one proteolytic fragment, the one with lower electrophoretic mobility observed in LAT-NIL expressing cells (Figure 4B, lanes 7 and 8). Conversely, Fas-mediated cleavage of LAT-NIL^D184A^ mutant generated only the lower band, indicating that the upper band is generated by cleavage at aspartic at position 184 of LAT-NIL, and the lower band is produced upon cleavage at Asp136. Overall these results show that the negatively charged stretch of LAT has a role in the stability of the molecule, without a role in the proteolytic cleavage of this membrane adapter.

### Analysis of Proximal TCR Intracellular Signals in LAT-NIL Expressing Cells

**Figure 5:**
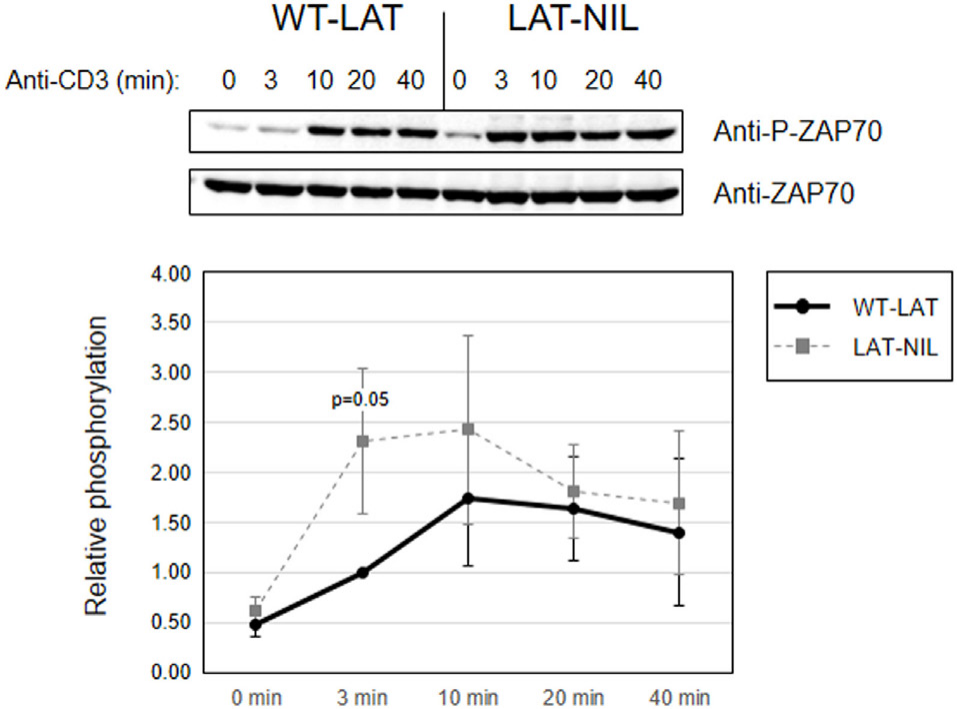
ZAP70 activation is increased in linker for activation of T cell (LAT)-NIL expressing cells. Phosphorylation of ZAP70 in WT-LAT or LAT-NIL expressing cells stimulated with soluble anti-CD3 was detected by Western blot using antibodies against pY319-ZAP70 (upper panel) and total ZAP70 (lower panel). The lower diagram represents the quantification of six independent experiments using J.CaM2 cells expressing WT-LAT (thick continuous line) or the LAT-NIL mutant (thin dashed line). Phosphorylation levels were normalized to total ZAP70 expression and the means of fold increase of phosphorylation with regard to non-stimulated cells were graphed. Bars represent the standard error.

**Figure 6:**
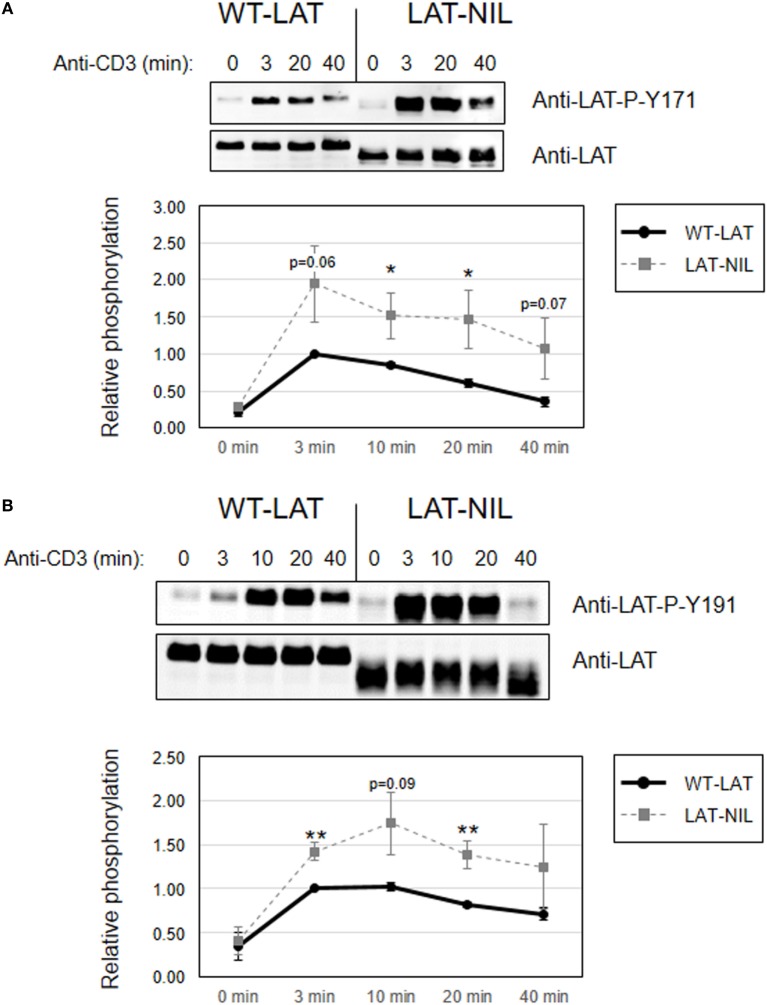
Increased phosphorylation of tyrosines 171 and 191 in linker for activation of T cell (LAT)-NIL expressing cells. Immunoblots analyzing phosphorylation of LAT at tyrosine residues 171 **(A)** or 191 **(B)** in cells stimulated with soluble anti-CD3 were done with phospho-specific antibodies. Membranes were stripped and blotted with anti-LAT antibody (middle panels). Lower diagrams represent the mean fold increase in phosphorylation in four **(A)** or six **(B)** independent experiments using J.CaM2 cells expressing WT-LAT (thick continuous line) or the LAT-NIL mutant (thin dashed line). Phosphorylation levels were normalized to total LAT expression. Bars represent the standard error. Asterisk represents statistical significance.

Since interaction of Lck with LAT downregulates its kinase activity (21), we next investigated early TCR signaling in LAT-NIL expressing cells. We were not able to directly detect activation-induced phosphorylation of Tyr394 of Lck in J.CaM2 cells expressing WT-LAT or the LAT-NIL mutant (data not shown), although this was not unexpected because it has been previously observed that anti-CD3 stimulation of Jurkat cells or primary human T cells does not induce substantial increase in the phosphorylation of this tyrosine residue (29, 30). So, considering that ZAP70 is an Lck kinase substrate (26) and constitutes one of the initial intracellular signals triggered after TCR engagement in T cells, we analyzed ZAP70 phosphorylation at tyrosine residue 319, which is required for its enzymatic activation (31). J.CaM2 cells expressing WT-LAT or LAT-NIL were stimulated with anti-CD3 antibodies, and cell lysates were analyzed by Western blot with a specific antibody for phosphorylated Tyr 319 of ZAP70 (Figure 5). As expected, CD3 stimulation of WT-LAT expressing cells induced a rapid increase in ZAP70 phosphorylation. Interestingly, activation-induced ZAP70 phosphorylation was increased in LAT-NIL with regard to WT-LAT expressing cells. Although our results only showed borderline statistical significance (*p* = 0.051 at 3 min), this result suggests a possible negative regulatory role of LAT–Lck interaction (21, 22). Provided that LAT is a ZAP70 substrate, we next analyzed LAT phosphorylation in lysates obtained in CD3-stimulated cells. LAT phosphorylation at tyrosine residues 171 and 191 (Figures 6A,B, respectively) was enhanced in LAT-NIL expressing cells, at all analyzed time points, and statistically significant differences were observed after 10 and 20 min of CD3 stimulation (Figure 6A). Also, at 3 and 40 min of stimulation the difference in Tyr171 phosphorylation between WT-LAT and LAT-NIL almost attained statistical significance (*p* = 0.059 and *p* = 0.071, respectively). We also analyzed phosphorylation of LAT tyrosine 191, and again we observed an increase in the phosphorylation of this residue in LAT-NIL, with statistical significance at 3 and 10 min time points (Figure 6B). Strikingly, blots of phospho-Tyr171 and phospho-Tyr191 show rather similar electrophoretic mobility, although the same membranes stripped and blotted with total anti-LAT antibody show the difference in the electrophoretic mobility already observed with anti-LAT and anti-6His antibodies (Figure 1B).

**Figure 7:**
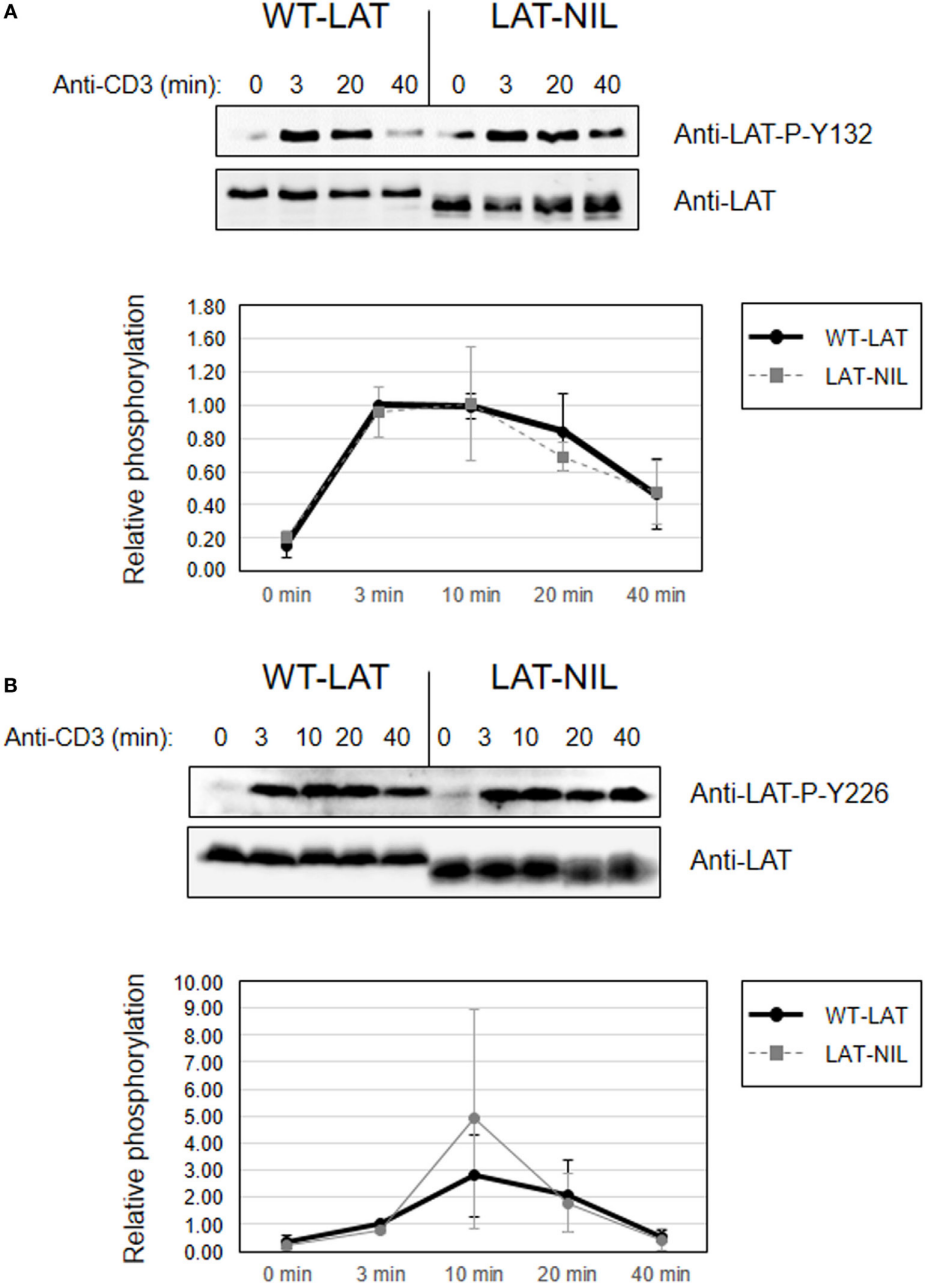
Phosphorylation of linker for activation of T-cell (LAT) tyrosines 132 and 226. Phosphorylation of LAT at tyrosine residues 132 **(A)** or 226 **(B)** in WT-LAT or LAT-NIL expressing cells. Membranes were stripped and blotted with anti-LAT antibody (middle panels). Lower diagrams represent the mean fold increase in phosphorylation in three independent experiments using J.CaM2 cells expressing WT-LAT (thick continuous line) or the LAT-NIL mutant (thin dashed line). Phosphorylation levels were normalized to total LAT expression.

Considering the proximity of the negatively charged fragment of amino acids substituted in LAT-NIL to tyrosine 132, we next analyzed the effect of this mutation on the phosphorylation of Tyr 132. Contrary to the results obtained with tyrosines 171 and 191, Western blot analysis performed in whole-cell lysates obtained from anti-CD3-stimulated cells expressing either WT-LAT or the LAT-NIL revealed no differences in the intensity or the kinetics of phosphorylation of Tyr 132 (Figure 7A). The same analysis was done for LAT tyrosine 226, and again there were no differences between WT-LAT and LAT-NIL (Figure 7B). Interestingly, as it happened in the analysis of phosphorylation of LAT tyrosines 171 and 191, we observed again the same behavior in the electrophoretic mobility of WT-LAT and LAT-NIL: membranes probed with phospho-specific antibodies showed a small decrease in the electrophoretic mobility of LAT-NIL with regard to WT-LAT, and after stripping and hybridization with anti-LAT antibody, the difference in electrophoretic mobility was increased. Given the defect observed in LAT-NIL stability after cycloheximide treatment (Figure 4A), we decided to verify LAT expression in cultured cells in order to exclude that the observed effects were not due to a diminished stability of LAT-NIL. Consequently, we performed Western blotting analysis of cell lysates obtained from J.CaM2 cells 1, 2, 3, and 4 weeks after lentiviral transduction with WT-LAT or LAT-NIL vectors. As it can be seen in Figure S2 in Supplementary Material, J.CaM2 cells lentivirally transduced with the LAT-NIL mutant stably expressed similar levels to WT-LAT, either with anti-LAT or anti-6His antibodies. Therefore, these results show that substitution of the negatively charged stretch of LAT has differential effects on the phosphorylation of its four functionally relevant tyrosines, affecting its electrophoretic mobility.

### Substitution of the Negatively Charged Stretch of LAT Negatively Affects Downstream Signals

**Figure 8:**
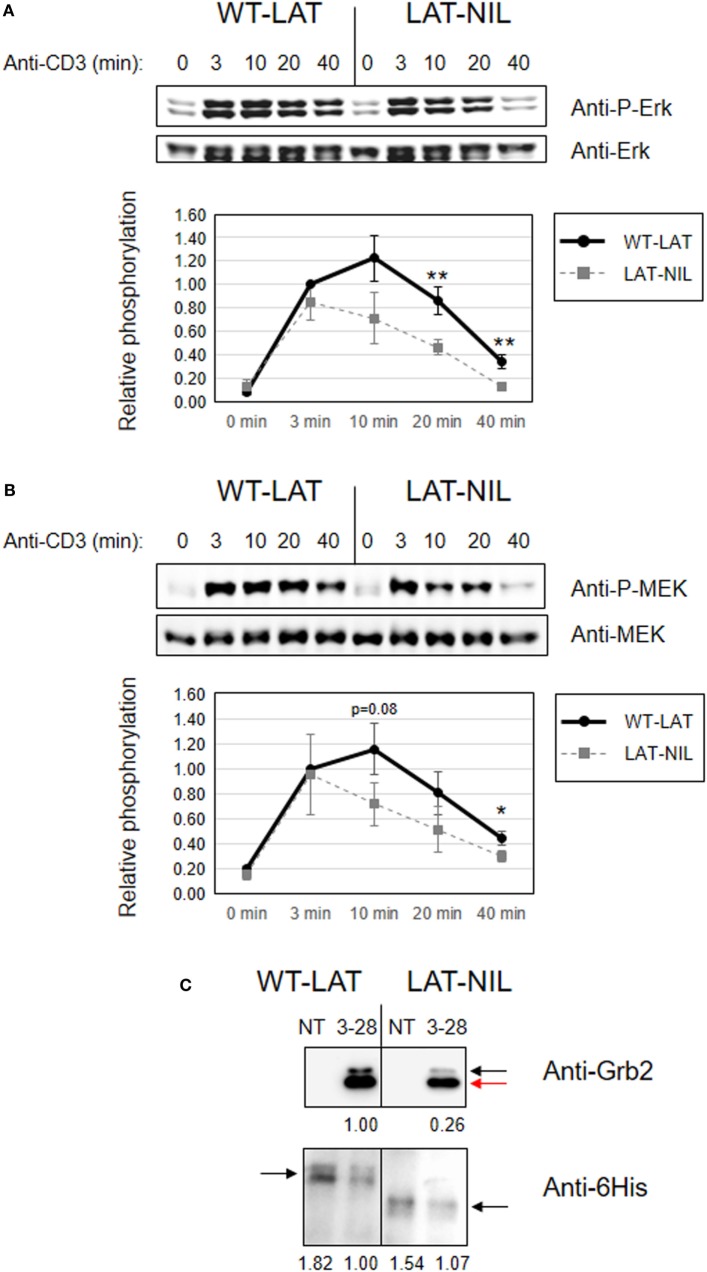
Negative impact of linker for activation of T cell (LAT)-NIL expression on MAPK pathway activation. JCaM2 cells expressing WT-LAT or the LAT-NIL mutant were stimulated at 37°C with anti-CD3 antibody for the indicated times. **(A)** Whole-cell lysates were probed by Western blotting for the activation of Erk by using a mAb recognizing doubly phosphorylated on specific threonine and tyrosine residues on Erk (upper panel). Stripped membranes were blotted with anti-pan Erk mAb to show equal protein expression (middle panel). Densitometric quantification of six independent experiments is shown in the lower diagram, representing mean fold increase in Erk phosphorylation for J.CaM2 cells expressing WT-LAT (thick continuous line) or the LAT-NIL mutant (thin dashed line). Phosphorylation levels were normalized to total Erk expression. **(B)** Whole-cell lysates were assayed for MEK1/2 activation with a mAb detecting specifically phosphorylation of Ser221. Stripped membranes were also blotted with anti-pan MEK1/2 mAb to show equal protein expression. Lower diagram represents the mean fold increase of MEK phosphorylation in five independent experiments. Bars represent the standard error. Asterisks represent statistical significance. **(C)** 10^7^ J.CaM2 cells expressing either WT-LAT or the LAT-NIL mutant were left untreated (NT) or stimulated for 5 min with 10 µg of anti-CD3 plus 10 µg of anti-CD28 antibodies, and postnuclear lysates were subjected to LAT immunoprecipitation with an anti-LAT antibody. The precipitates were analyzed by Western blot with an anti-Grb2 antibody (upper panel) or an anti-6His antibody (lower panel), as indicated. Images are representative of three independent experiments. Numbers below the blots indicate the densitometric quantification of the corresponding bands. Black arrow in the upper blot indicates the band corresponding to Grb2, and the red arrow indicates the position of the light chains. Black arrows in the lower blot indicate the position of LAT.

Considering the differential effects induced by the substitution of the negatively charged stretch of LAT in the phosphorylation of its tyrosine residues, we were then interested in the analysis of downstream signals. Previous reports had shown the negative impact of mutation of tyrosines 171 and 191 in TCR downstream signals (6, 11, 32). Thus, the increased phosphorylation of these residues observed in LAT-NIL should induce an increase in downstream signals. Both tyrosine residues have an identical Grb2-binding motif (YVNV), a small cytosolic adaptor consisting in two SH3 domains and one SH2 domain, which binds to phosphorylated tyrosines 171, 191, and 226 in LAT (6, 33). Grb2 constitutively binds through its SH3 domains to a proline-rich region in the Ras guanine nucleotide exchange factor, SOS, and phosphorylation of LAT recruits SOS close to Ras at the cell membrane, allowing the activation of the MAPK pathway (34). Therefore, we analyzed phosphorylation of Erk at Thr202 and Tyr204 in WT-LAT and LAT-NIL expressing cells, since phosphorylation of these two residues is indicative of Erk enzymatic activation (35). As expected, anti-CD3 stimulation of J.CaM2 cells expressing WT-LAT induced a rapid increase in Erk phosphorylation after 3 min (Figure 8A). LAT-NIL expressing cells showed an initial similar increase in activation-induced Erk phosphorylation after 3 min of stimulation, although at longer time points the kinetics were different, with LAT-NIL showing a slightly reduced phosphorylation. However, we did not find statistically significant differences between Erk phosphorylation in WT-LAT and LAT-NIL expressing cells. In order to go deeper into the analysis of the effects of LAT-NIL mutant in the MAPK pathway, we analyzed activation of MEK, the kinase responsible of the phosphorylation and activation of Erk (36). Therefore, we used a specific antibody to phospho-MEK-1/2 to investigate MEK activation. CD3 stimulation of WT-LAT expressing cells rapidly induced phosphorylation of MEK and the same was observed for LAT-NIL expressing cells (Figure 8B). However, at longer time points, LAT-NIL expressing cells showed a faster decay in the phosphorylation level of MEK, with regard to WT-LAT expressing cells, similar to Erk phosphorylation kinetics. Densitometric quantification of five independent experiments showed that the substitution of negatively charged fragment between residues 112 and 126 of LAT had a negative impact on MEK phosphorylation, with statistical significance at 10 and 40 min time points (Figure 8B, lower graphics). Therefore, in contrast to the increase in early intracellular signals, more distal signals such as Erk and MEK activation are negatively affected by the substitution of the negatively charged stretch in LAT. Next, in order to assess if the observed defects were due to a decreased ability of LAT-NIL mutant to bind to Grb2, we immunoprecipitated LAT after anti-CD3 plus anti-CD28 stimulation of lentivirally transduced JCaM2.5 cells expressing either WT-LAT or LAT-NIL. Western blot analysis showed a reduced recruitment of Grb2 by LAT-NIL versus WT-LAT, although the amount of both precipitated forms of LAT were comparable (Figure 8C).

Next, in light of the negative effects of LAT-NIL mutation in MAPK pathway activation, we speculated if TCR-mediated induction of CD69 expression was affected (37). J.CaM2 cells expressing WT-LAT and LAT-NIL were stimulated for 18 h with immobilized anti-CD3 antibodies, and CD69 was analyzed by flow cytometry. Anti-CD3 stimulation induced moderate levels of CD69 expression in WT-LAT expressing cells (14.2%), although CD69 percentage was always lower in LAT-NIL cells (7.4%), with statistical significance difference in three independent experiments (Figure S3 in Supplementary Material). Thus, the negatively charged domain of LAT is needed for a proper activation of the MAPK pathway, and hence, the TCR-mediated induction of CD69 expression.

**Figure 9:**
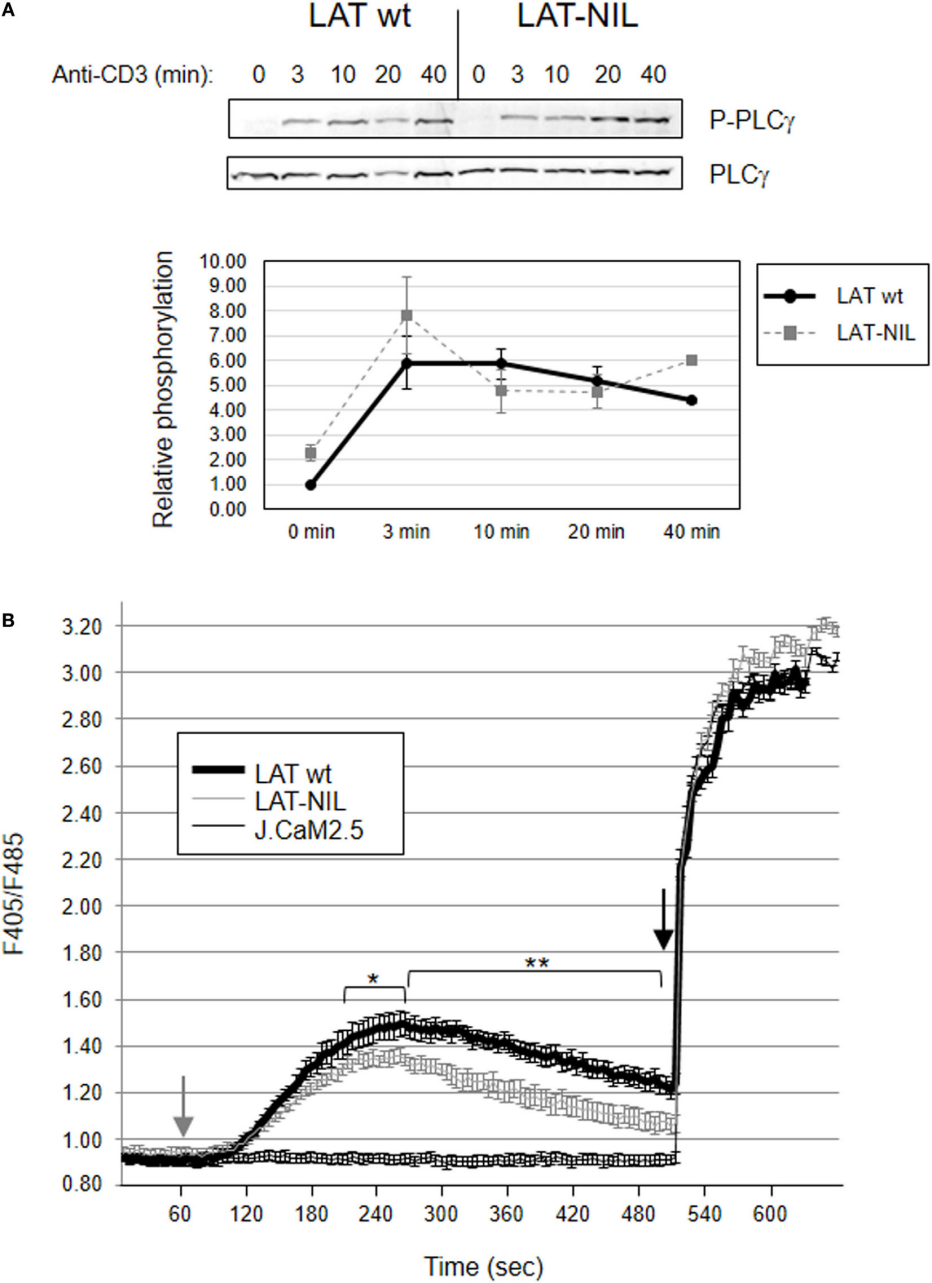
Activation of PLC-γ and Ca^2+^ influx generation. **(A)** JCaM2 cells expressing WT-linker for activation of T cell (LAT) or the LAT-NIL mutant were stimulated at 37°C with anti-CD3 antibody for the indicated times, and whole-cell lysates were probed by Western blotting for the activation of PLC-γ1 by using a mAb recognizing PLC-γ1 phosphorylated on tyrosine 783 (upper panel). Stripped membranes were blotted with anti-PLC-γ1 mAb to show equal protein expression (middle panel). Densitometric quantification of six independent experiments is shown in the lower diagram, representing mean fold increase in PLC-γ1 phosphorylation for J.CaM2 cells expressing WT-LAT (thick continuous line) or the LAT-NIL mutant (thin dashed line). **(B)** Untransduced J.CaM2 cells or J.CaM2 cells expressing WT-LAT or the LAT-NIL mutant were loaded with Indo-1AM and stimulated with OKT3 mAb (1 µg/ml) at the indicated time (grey arrow). The intracellular Ca^2+^ concentration was determined at 37°C through the change in Indo-1AM fluorescence. The charge of Indo-1AM dye was assessed by observing the response to stimulation with ionomycin (1 μM, black arrow). Bars represent the standard error. Asterisks represent statistical significance.

We next analyzed the effect of replacing the fragment between residues 112 and 126 of LAT on PLC-γ1 activation. TCR ligation leads to rapid LAT phosphorylation, thereby allowing the recruitment of PLC-γ1 to the cellular membrane, where it is phosphorylated on Tyr783, a hallmark of its enzymatic activation (38). Anti-CD3 stimulation induced phosphorylation of PLC-γ1 in both WT-LAT and LAT-NIL J.CaM2 expressing cells (Figure 9A). The kinetics were similar in both types of cells, although there was a slightly enhanced increase in PLC-γ phosphorylation in LAT-NIL expressing cells, but without statistically significant differences. This observation was consistent with the similar intensity and kinetics of phosphorylation of LAT tyrosine 132, the principal binding site for PLC-γ1. Consequently, we next analyzed whether the substitution of the negatively charged fragment of LAT had an effect on calcium influx generation. J.CaM2 cells are deficient in TCR-mediated intracellular calcium generation (8), and lentiviral expression of the WT form of LAT restored Ca^2+^ influx (Figure 9B). CD3 stimulation of LAT-NIL expressing cells also restored the increase in cytoplasmic Ca^2+^ concentration, with initial kinetics very similar to WT-LAT expressing cells. However, J.CaM2 cells expressing the LAT-NIL mutant had a statistical significant reduction in both the peak and the sustained levels of calcium influx. Therefore, these results show the calcium influx is negatively affected by the substitution of the negatively charged stretch of LAT. Although similar kinetics in PLC-γ1 phosphorylation and reduced Ca^2+^ influx seem contradictory, it has been recently observed that phosphorylation of PLC-γ1 is initiated very quickly after TCR stimulation, similar to LAT tyrosine 132; however, 20 and 30 min after stimulation a significant portion PLC-γ1 is still phosphorylated, but phosphorylation of LAT Tyr132 has recovered basal levels (39). These data suggest that the current model of PLC-γ1 activation is incomplete, and although phosphorylation of LAT at Tyr132 is needed for the initial binding of PLC-γ1, this interaction is transient and other signals regulate PLC-γ1 activation at longer time points. Our results support this report, and substitution of sequence between amino acids 112 and 126 in LAT generates diminished calcium mobilization without appreciable differences in LAT-Tyr132 phosphorylation.

## Discussion

The integral membrane adaptor LAT has a crucial role in TCR-mediated activation of T cells, and its five C-terminal tyrosine residues are needed for the transduction of signals leading to cell activation (1). However, data obtained from the analysis of several strains of mice expressing different mutant forms of this adaptor have revealed that LAT has an intrinsic ability to regulate T-cell homeostasis, although the molecular basis for this negative role is not completely understood (14–17, 40). A self-explanatory negative feedback loop involving LAT was described in 2004 by Dr Koyasu’s group, by which TCR-mediated activation would lead to LAT-dependent activation of Erk, which in turn was able to phosphorylate human LAT at Thr155, thereby reducing the ability of LAT to recruit PLC-γ1 and decreasing Ca^2+^ mobilization and MAPK pathway activation (41). This negative feedback loop would prevent TCR signaling with inappropriate duration or intensity, which could be potentially harmful for the immune system, and failures of this regulation mechanism could be at the base of immune disorders. However, this threonine residue is not conserved in other species like mouse or rat, excluding an essential role for Thr155 in the negative functions played by LAT in these species and preventing *in vivo* analysis of the role played by such feedback loop.

In this context, it has been previously described that upon TCR-mediated activation of T cells, LAT interacts with Lck and this interaction decreases Lck kinase activity (21, 23). Very interestingly, by means of expressing truncated forms of LAT, it was shown that a truncated form of LAT at Asp126 was still able to interact with Lck but not an isoform truncated at Asn112 (22). Therefore, LAT–Lck interaction could constitute a model for termination of activatory signals coming from the TCR/CD3 complex. The fragment between residues 112 and 126 in human LAT is composed by a stretch of negatively charged amino acids, and this sequence is evolutionarily conserved in human, mouse, rat, gorilla, chimpanzee, cow, cat, and other species, supporting an important role for this fragment of LAT for its functions in intracellular signaling coupled to the TCR/CD3 complex (see Table 1). Remarkably, this fragment is preceding the most N-terminal cleavage point of human LAT (18), and Fas-mediated cleavage at this point would generate a LAT fragment still able to bind to Lck and diminishing its kinase activity (21, 22). This prompted us to analyze the role of this stretch by means of substituting it with a flexible peptide fragment without negatively charged amino acids. Our results confirm that this sequence of LAT is necessary for the activation-induced interaction of LAT with Lck kinase, since the LAT-NIL mutant did not show the increase of LAT-Lck interaction previously described upon PHA or pervanadate stimulation (22, 23, 42), contrary to WT-LAT. Moreover, we have shown that LAT–Lck interaction constitutes a regulatory mechanism for the TCR signaling cassette, since the mutation of the negatively charged stretch of amino acids in LAT increases the TCR-mediated phosphorylation of Tyr319 in the interdomain B of ZAP70, required for the activation of ZAP70 function (31). We were not able to directly detect activation-induced phosphorylation of Tyr394 of Lck in J.CaM2 cells expressing WT-LAT or the LAT-NIL mutant (data not shown), although this was not unexpected because it has been previously observed that anti-CD3 stimulation of Jurkat cells or primary human T cells does not induce substantial increase in the phosphorylation of this tyrosine residue (29, 30).

Consistent with the increase in ZAP70 activation, anti-CD3 stimulation of LAT-NIL expressing cells induced augmented phosphorylation of LAT tyrosines 171 and 191 with regard to WT-LAT expressing cells. However, tyrosine residues 132 and 226 showed a different behavior, with similar levels of phosphorylation in the basal state and at different time points after TCR stimulation. This was not completely unexpected, since there are multiple evidences of functional specialization of the last five C-terminal tyrosine residues of LAT (11, 15, 32, 43). Moreover, two independent groups have previously demonstrated that tyrosine residues 132 and 191 have different kinetics of phosphorylation, with a delay in the phosphorylation of Tyr132 with regard to Tyr191 (39, 44). Strikingly, we have observed an increase of phosphorylation of both Tyr171 and Tyr191 in LAT and Tyr319 of ZAP70 in LAT-NIL expressing cells. Increase in phosphorylation of Tyr171 as a consequence of augmented activity of ZAP70 in LAT-NIL expressing cells is also consistent with a recent report by Kuriyan and co-workers (26). Kuriyan’s group has used an *in vitro* approach to identify peptide substrates for ZAP70 and Lck, showing that tyrosine 191 in LAT is a relatively poor substrate for ZAP70, which is not in agreement with the results presented in this work. Nevertheless, we cannot discard that phosphorylation efficiency in the whole LAT molecule, attached to the membrane, can be somewhat different to the phosphorylation of peptides bound to the surface of bacteria. Moreover, it has been recently shown that LFA crosslinking induces phosphorylation of LAT at tyrosine 171 by Focal Adhesion Kinase 1 (FAK1), and this event leads to a reduction in proliferation and adhesion of T cells to dendritic cells (45). This is consistent with our results, since the mutation introduced in LAT-NIL increased phosphorylation of Tyr171, leading to a reduction of downstream intracellular signals. More work is needed to clarify the molecular mechanisms underlying this observation.

In contrast to the increase in early intracellular signals, more distal signals such as the MAPK pathway activation or the increase in the calcium concentration are negatively affected by the substitution of the negatively charged stretch in LAT. LAT-NIL expressing cells show a statistically significant reduction in the phosphorylation of Thr202 and Tyr204 in Erk, which is indicative of its enzymatic activation (35). This result was in agreement with the reduction of phosphorylation of MEK, the kinase responsible of Erk phosphorylation. Indeed we have observed a strikingly similar kinetics for both Erk and MEK phosphorylation, with a rapid and very similar increase in phosphorylation at short time points in WT-LAT and LAT-NIL expressing cells (3 min), and a reduction in phosphorylation at longer time points in LAT-NIL expressing cells (more than 10 min). In the same way, although LAT-NIL expressing cells rapidly increased calcium concentration after CD3 stimulation, they showed a significant reduction in both the peak and the sustained levels of calcium influx with respect to WT-LAT expressing cells. However, PLC-γ1 phosphorylation at Tyr783, indicative of its enzymatic activation, was similar in both types of cells (with a slightly enhanced increase in LAT-NIL expressing cells). We have also seen no differences in the phosphorylation of LAT tyrosine 132, the binding point of PLC-γ1. Although both observations seem inconsistent, it has been recently reported that even if initial activation of PLC-γ1 requires LAT-Y132 phosphorylation, once triggered, PLC-γ1 activation is independent of LAT and LAT and activated PLC-γ are located in different membrane clusters (39), suggesting that although phosphorylation of LAT at Tyr132 is needed for the initial binding and activation of PLC-γ1, this interaction is transient and other signals are needed to keep PLC-γ1 activated at longer time points. Our results are in agreement with these observations, and although the substitution of sequence between amino acids 112 and 126 in LAT does not appreciably modify phosphorylation of Tyr132, a statistically significant decrease in calcium mobilization is observed a long times (Figure 9B). One possible explanation for these results could be that the imbalance generated by the increased tyrosine phosphorylation of Tyr171 and Tyr191 could lead to diminished activation of the MAPK pathway, calcium mobilization or CD69 expression, as it happens in two LAT-KI strains of mice (14–16). Another possible explanation for the decrease in downstream signaling is the reduced ability of Grb2 recruitment of LAT-NIL mutant, which is in agreement with a previous report showing that Grb2 deficient cells have reduced MAP kinase activation and calcium flux (46).

It is also of interest the reduction observed in the stability of LAT-NIL mutant with regard to the WT isoform. This result is consistent with our previous observation of a LAT isoform resulting from an intron 6 retention event, which generates an in-frame splice denoted as LAT_i6_ (28). The amino acid sequence introduced as a consequence of the retention of intron 6 is located just after Glu113, at the beginning of the negatively charged stretch of LAT needed for the interaction with Lck, confirming a relevant role for this part of LAT in its stability and/or turnover. Interestingly, it seems that the decreased interaction with Lck could be at the base of this reduction of LAT-NIL stability, since Lck deficient J.CaM1.6 cells express basal lower levels of LAT with regard to Jurkat cells (from which they are derived) (47). Moreover, the stability of LAT in J.CaM1.6 cells after cycloheximide treatment is clearly reduced with regard to LAT in Jurkat cells. Although it has been previously shown that the lack of LAT expression in J.CaM2 cells decreases Lck enzymatic activity (21), to our knowledge this is the first time that a reduction of LAT expression in Lck-deficient cells has been reported. Therefore, our data support a functional relationship between LAT and Lck, with Lck regulating LAT expression and LAT regulating Lck activity. Additional work should be done in order to reach a deeper understanding about this functional relationship.

Additionally, we have observed a change in electrophoretic mobility when the negatively charged stretch of LAT is substituted by a sequence of 12 amino acids with a net charge of +1, two residues shorter. We decided to introduce a shorter sequence in order to easily discriminate between the two isoforms, but in our view the difference in electrophoretic mobility is greater than expected. Moreover, Western blots with anti-LAT phospho-specific antibodies showed smaller differences in electrophoretic mobility than anti-total LAT or anti-6His antibodies in the same stripped membranes. It has been demonstrated that the p36/38 doublet is not due to alternative splicing, but probably to post-translational modifications of unknown nature. Although we have no explanation for this change in the mobility of LAT-NIL isoform, we think that phosphorylation differences are not at the basis of this event, since the basal phosphorylation state of tyrosine residues 132, 171, 191, and 226 is similar between WT and the LAT-NIL mutant, and calf intestinal phosphatase treatment did not modify electrophoretic mobility of either WT-LAT or LAT-NIL isoforms (data not shown).

In summary, the results presented extend previous reports about the role of LAT as an intrinsic regulator or terminator of intracellular signals associated to the TCR/CD3 complex. CD95 ligation in naive T cells interferes with proximal intracellular signals, probably by means of proteolytic cleavage of essential proteins in the signaling cassette (18, 20, 48–50). In this way, it is conceivable a scenario in which activated T cells expressing Fas interact with FasL expressing APCs and this interaction would induce LAT cleavage, preventing the transduction of high intensity or very prolonged activation signals. However, the TCR/CD3 complex is able to transduce intracellular signals in a LAT-independent way (51), and LAT cleavage would constitute an incomplete terminator mechanism. Although our study has the limitations due to the use of a cell line rather than primary T cells, our data suggest that Fas-mediated cleavage of LAT generates a membrane attached LAT isoform, still able to bind to Lck, inhibiting its kinase activity for a more complete termination of signals, with the negatively charged stretch of LAT having an essential role in this regulatory interaction.

## Ethics Statement

This study was carried out in accordance with the recommendations of the University of Cádiz and Puerto Real University Hospital ethics committees.

## Author Contributions

All authors contributed to discussions of experimental design and data analysis. MA-E, IN-S, and EA designed and performed most experiments and interpreted results. MA-E and EA wrote the manuscript. CF-P and IV-B provided technical assistance with transfections, cell culture and confocal microscopy. MD-R and IV-B provided assistance with Western blotting. MD, MR-Y, FG-C, MD-R, and AM provided input on study design and helped with manuscript writing. EA directed the study.

## Conflict of Interest Statement

The authors have no conflict of interest relative to the data reported in this manuscript.

## Supplementary Material

Figure S1 Stability of WT-linker for activation of T cell (LAT) and LAT-NIL mutant proteins. **(A)** J.CaM2 cells expressing WT-LAT or the LAT-NIL mutant were treated with cycloheximide (CHX) for indicated times in hours, and LAT and beta-actin protein levels were detected by Western blot. **(B)** Jurkat cells or the Lck-deficient cell line J.CaM1.6 were treated with cycloheximide (CHX) for indicated times in hours, and LAT (upper panels) and beta-actin (lower panels) protein levels were detected by Western blot. Images are representative of three independent experiments with similar results. Numbers below the blots indicate the densitometric quantification of the corresponding bands.

Figure S2 Expression of WT-linker for activation of T cell (LAT) and LAT-NIL mutant proteins. J.CaM2 cells were lentivirally transduced to express WT-LAT or the LAT-NIL mutant, and the expression of LAT was analyzed 1, 2, 3, and 4 weeks after lentiviral transduction by Western blotting with anti-LAT antibody or anti-6His antibody. Numbers below the blots indicate the densitometric quantification of the corresponding bands. Numbers below the blots indicate the densitometric quantification of the corresponding bands.

Figure S3 Negative impact of linker for activation of T cell (LAT)-NIL expression on activation-induced CD69 expression. Untransduced J.CaM2 cells or transduced with lentiviral vectors coding for WT-LAT or LAT-NIL were stimulated with immobilized anti-CD3 for 18 h at 37°C, and CD69 expression was analyzed by flow cytometry. Left and middle histograms show the result of a representative experiment showing CD69 expression (black line) in WT-LAT and LAT-NIL expressing cells. Gray lines indicate isotype-matched negative control antibody staining. Right panel shows average percentages of CD69^+^ cells in three independent experiments. Bars represent the standard error. Asterisks represent statistical significance.

## References

[1] Malissen B Bongrand P. Early T cell activation: integrating biochemical, structural, and biophysical cues. Annu Rev Immunol (2015) 33:539–61. 10.1146/annurev-immunol-032414-112158 25861978

[2] Mayya V Dustin ML. What scales the T cell response? Trends Immunol (2016) 37(8):513–22. 10.1016/j.it.2016.06.005 27364960

[3] Sykulev Y T cell receptor signaling kinetics takes the stage. Sci Signal (2010) 3(153):e50 10.1126/scisignal.3153pe50 PMC3040109 21177491

[4] Malissen B Aguado E Malissen M. Role of the LAT adaptor in T-cell development and Th2 differentiation. Adv Immunol (2005) 87:1–25. 10.1016/S0065-2776(05)87001-4 16102570

[5] Aguado E Martinez-Florensa M Aparicio P. Activation of T lymphocytes and the role of the adapter LAT. Transpl Immunol (2006) 17(1):23–6. 10.1016/j.trim.2006.09.013 17157209

[6] Zhang W Sloan-Lancaster J Kitchen J Trible RP Samelson LE LAT: the ZAP-70 tyrosine kinase substrate that links T cell receptor to cellular activation. Cell (1998) 92(1):83–92. 10.1016/S0092-8674(00)80901-0 9489702

[7] Weber JR Orstavik S Torgersen KM Danbolt NC Berg SF Ryan JC Molecular cloning of the cDNA encoding pp36, a tyrosine-phosphorylated adaptor protein selectively expressed by T cells and natural killer cells. J Exp Med (1998) 187(7):1157–61. 10.1084/jem.187.7.1157 9529333 PMC2212210

[8] Finco TS Kadlecek T Zhang W Samelson LE Weiss A LAT is required for TCR-mediated activation of PLCgamma1 and the Ras pathway. Immunity (1998) 9(5):617–26. 10.1016/S1074-7613(00)80659-7 9846483

[9] Zhang W Irvin BJ Trible RP Abraham RT Samelson LE. Functional analysis of LAT in TCR-mediated signaling pathways using a LAT-deficient Jurkat cell line. Int Immunol (1999) 11(6):943–50. 10.1093/intimm/11.6.943 10360968

[10] Zhang W Sommers CL Burshtyn DN Stebbins CC DeJarnette JB Trible RP Essential role of LAT in T cell development. Immunity (1999) 10(3):323–32. 10.1016/S1074-7613(00)80032-1 10204488

[11] Zhang W Trible RP Zhu M Liu SK McGlade CJ Samelson LE Association of Grb2, Gads, and phospholipase C-gamma 1 with phosphorylated LAT tyrosine residues. Effect of LAT tyrosine mutations on T cell angigen receptor-mediated signaling. J Biol Chem (2000) 275(30):23355–61. 10.1074/jbc.M000404200 10811803

[12] Paz PE Wang S Clarke H Lu X Stokoe D Abo A. Mapping the Zap-70 phosphorylation sites on LAT (linker for activation of T cells) required for recruitment and activation of signalling proteins in T cells. Biochem J (2001) 356(Pt 2):461–71. 10.1042/bj3560461 11368773 PMC1221857

[13] Sommers CL Menon RK Grinberg A Zhang W Samelson LE Love PE. Knock-in mutation of the distal four tyrosines of linker for activation of T cells blocks murine T cell development. J Exp Med (2001) 194(2):135–42. 10.1084/jem.194.2.135 11457888 PMC2193454

[14] Aguado E Richelme S Nunez-Cruz S Miazek A Mura AM Richelme M Induction of T helper type 2 immunity by a point mutation in the LAT adaptor. Science (2002) 296(5575):2036–40. 10.1126/science.1069057 12065839

[15] Sommers CL Park CS Lee J Feng C Fuller CL Grinberg A A LAT mutation that inhibits T cell development yet induces lymphoproliferation. Science (2002) 296(5575):2040–3. 10.1126/science.1069066 12065840

[16] Nunez-Cruz S Aguado E Richelme S Chetaille B Mura AM Richelme M LAT regulates gammadelta T cell homeostasis and differentiation. Nat Immunol (2003) 4(10):999–1008. 10.1038/ni977 12970761

[17] Mingueneau M Roncagalli R Gregoire C Kissenpfennig A Miazek A Archambaud C Loss of the LAT adaptor converts antigen-responsive T cells into pathogenic effectors that function independently of the T cell receptor. Immunity (2009) 31(2):197–208. 10.1016/j.immuni.2009.05.013 19682930

[18] Garcia-Blesa A Klossowicz M Lopez-Osuna C Martinez-Florensa M Malissen B Garcia-Cozar FJ The membrane adaptor LAT is proteolytically cleaved upon Fas engagement in a tyrosine phosphorylation dependent fashion. Biochem J (2013) 450(3):511–21. 10.1042/BJ20121135 23240581

[19] Klossowicz M Scirka B Suchanek J Marek-Bukowiec K Kisielow P Aguado E Assessment of caspase mediated degradation of linker for activation of T cells (LAT) at a single cell level. J Immunol Methods (2013) 389(1–2):9–17. 10.1016/j.jim.2012.12.004 23261919

[20] Arbulo-Echevarria MM Munoz-Miranda JP Caballero-Garcia A Poveda-Diaz JL Fernandez-Ponce C Duran-Ruiz MC Non-T cell activation linker (NTAL) proteolytic cleavage as a terminator of activatory intracellular signals. J Leukoc Biol (2016) 100(2):351–60. 10.1189/jlb.2A0715-318R 26830332

[21] Kabouridis PS. Selective interaction of LAT (linker of activated T cells) with the open-active form of Lck in lipid rafts reveals a new mechanism for the regulation of Lck in T cells. Biochem J (2003) 371(Pt 3):907–15. 10.1042/BJ20021578 12570875 PMC1223349

[22] Kabouridis PS Isenberg DA Jury EC A negatively charged domain of LAT mediates its interaction with the active form of Lck. Mol Membr Biol (2011) 28(7–8):487–94. 10.3109/09687688.2011.624990 22034845

[23] Jiang Y Cheng H. Evidence of LAT as a dual substrate for Lck and Syk in T lymphocytes. Leuk Res (2007) 31(4):541–5. 10.1016/j.leukres.2006.07.010 16938345

[24] Huang F Nau WM A conformational flexibility scale for amino acids in peptides. Angew Chem Int Ed Engl (2003) 42(20):2269–72. 10.1002/anie.200250684 12772159

[25] Lee GM Fong SS Oh DJ Francis K Palsson BO. Characterization and efficacy of PKH26 as a probe to study the replication history of the human hematopoietic KG1a progenitor cell line. In Vitro Cell Dev Biol Anim (2002) 38(2):90–6. 10.1290/1071-2690(2002)038<0090:CAEOPA>2.0.CO;2 11929001

[26] Shah NH Wang Q Yan Q Karandur D Kadlecek TA Fallahee IR An electrostatic selection mechanism controls sequential kinase signaling downstream of the T cell receptor. Elife (2016) 5:e20105. 10.7554/eLife.20105 27700984 PMC5089863

[27] Dong S Corre B Nika K Pellegrini S Michel F. T cell receptor signal initiation induced by low-grade stimulation requires the cooperation of LAT in human T cells. PLoS One (2010) 5(11):e15114. 10.1371/journal.pone.0015114 21152094 PMC2994893

[28] Klossowicz M Marek-Bukowiec K Arbulo-Echevarria MM Scirka B Majkowski M Sikorski AF Identification of functional, short-lived isoform of linker for activation of T cells (LAT). Genes Immun (2014) 15(7):449–56. 10.1038/gene.2014.35 25008862

[29] Nika K Soldani C Salek M Paster W Gray A Etzensperger R Constitutively active Lck kinase in T cells drives antigen receptor signal transduction. Immunity (2010) 32(6):766–77. 10.1016/j.immuni.2010.05.011 20541955 PMC2996607

[30] Stirnweiss A Hartig R Gieseler S Lindquist JA Reichardt P Philipsen L T cell activation results in conformational changes in the Src family kinase Lck to induce its activation. Sci Signal (2013) 6(263):ra13. 10.1126/scisignal.2003607 23423439

[31] Di Bartolo V Mege D Germain V Pelosi M Dufour E Michel F Tyrosine 319, a newly identified phosphorylation site of ZAP-70, plays a critical role in T cell antigen receptor signaling. J Biol Chem (1999) 274(10):6285–94. 10.1074/jbc.274.10.6285 10037717

[32] Lin J Weiss A. Identification of the minimal tyrosine residues required for linker for activation of T cell function. J Biol Chem (2001) 276(31):29588–95. 10.1074/jbc.M102221200 11395491

[33] Zhu M Janssen E Zhang W. Minimal requirement of tyrosine residues of linker for activation of T cells in TCR signaling and thymocyte development. J Immunol (2003) 170(1):325–33. 10.4049/jimmunol.170.1.325 12496416

[34] Wange RL LAT, the linker for activation of T cells: a bridge between T cell-specific and general signaling pathways. Sci STKE (2000) 2000(63):re1 10.1126/stke.2000.63.re1 11752630

[35] Payne DM Rossomando AJ Martino P Erickson AK Her JH Shabanowitz J Identification of the regulatory phosphorylation sites in pp42/mitogen-activated protein kinase (MAP kinase). EMBO J (1991) 10(4):885–92. 1849075 10.1002/j.1460-2075.1991.tb08021.x PMC452730

[36] Crews CM Alessandrini A Erikson RL. The primary structure of MEK, a protein kinase that phosphorylates the ERK gene product. Science (1992) 258(5081):478–80. 10.1126/science.1411546 1411546

[37] D’Ambrosio D Cantrell DA Frati L Santoni A Testi R. Involvement of p21ras activation in T cell CD69 expression. Eur J Immunol (1994) 24(3):616–20. 10.1002/eji.1830240319 7907294

[38] Wang Z Gluck S Zhang L Moran MF. Requirement for phospholipase C-gamma1 enzymatic activity in growth factor-induced mitogenesis. Mol Cell Biol (1998) 18(1):590–7. 10.1128/MCB.18.1.590 9418905 PMC121526

[39] Cruz-Orcutt N Vacaflores A Connolly SF Bunnell SC Houtman JC Activated PLC-gamma1 is catalytically induced at LAT but activated PLC-gamma1 is localized at both LAT- and TCR-containing complexes. Cell Signal (2014) 26(4):797–805. 10.1016/j.cellsig.2013.12.022 24412752 PMC3935424

[40] Wang Y Kissenpfennig A Mingueneau M Richelme S Perrin P Chevrier S Th2 lymphoproliferative disorder of LatY136F mutant mice unfolds independently of TCR-MHC engagement and is insensitive to the action of Foxp3+ regulatory T cells. J Immunol (2008) 180(3):1565–75. 10.4049/jimmunol.180.3.1565 18209052

[41] Matsuda S Miwa Y Hirata Y Minowa A Tanaka J Nishida E Negative feedback loop in T-cell activation through MAPK-catalyzed threonine phosphorylation of LAT. EMBO J (2004) 23(13):2577–85. 10.1038/sj.emboj.7600268 15192708 PMC449778

[42] Sturgill TW Ray LB Erikson E Maller JL. Insulin-stimulated MAP-2 kinase phosphorylates and activates ribosomal protein S6 kinase II. Nature (1988) 334(6184):715–8. 10.1038/334715a0 2842685

[43] Zhang W Trible RP Samelson LE LAT palmitoylation: its essential role in membrane microdomain targeting and tyrosine phosphorylation during T cell activation. Immunity (1998) 9(2):239–46. 10.1016/S1074-7613(00)80606-8 9729044

[44] Houtman JC Houghtling RA Barda-Saad M Toda Y Samelson LE. Early phosphorylation kinetics of proteins involved in proximal TCR-mediated signaling pathways. J Immunol (2005) 175(4):2449–58. 10.4049/jimmunol.175.4.2449 16081816 PMC1414060

[45] Raab M Lu Y Kohler K Smith X Strebhardt K Rudd CE LFA-1 activates focal adhesion kinases FAK1/PYK2 to generate LAT-GRB2-SKAP1 complexes that terminate T-cell conjugate formation. Nat Commun (2017) 8:16001 10.1038/ncomms16001 28699640 PMC5510181

[46] Bilal MY Houtman JC GRB2 nucleates T cell receptor-mediated LAT clusters that control PLC-gamma1 activation and cytokine production. Front Immunol (2015) 6:141 10.3389/fimmu.2015.00141 25870599 PMC4378308

[47] Straus DB Weiss A. Genetic evidence for the involvement of the Lck tyrosine kinase in signal transduction through the T cell antigen receptor. Cell (1992) 70(4):585–93. 10.1016/0092-8674(92)90428-F 1505025

[48] Yankee TM Draves KE Ewings MK Clark EA Graves JD. CD95/Fas induces cleavage of the GrpL/Gads adaptor and desensitization of antigen receptor signaling. Proc Natl Acad Sci U S A (2001) 98(12):6789–93. 10.1073/pnas.111158598 11391000 PMC34431

[49] Berry DM Benn SJ Cheng AM McGlade CJ. Caspase-dependent cleavage of the hematopoietic specific adaptor protein Gads alters signalling from the T cell receptor. Oncogene (2001) 20(10):1203–11. 10.1038/sj.onc.1204218 11313864

[50] Strauss G Lindquist JA Arhel N Felder E Karl S Haas TL CD95 co-stimulation blocks activation of naive T cells by inhibiting T cell receptor signaling. J Exp Med (2009) 206(6):1379–93. 10.1084/jem.20082363 19487421 PMC2715064

[51] Roncagalli R Hauri S Fiore F Liang Y Chen Z Sansoni A Quantitative proteomics analysis of signalosome dynamics in primary T cells identifies the surface receptor CD6 as a Lat adaptor-independent TCR signaling hub. Nat Immunol (2014) 15(4):384–92. 10.1038/ni.2843 24584089 PMC4037560

