# Supplementary figures and images for: A Stretch of Negatively Charged Amino Acids of Linker for Activation of T-Cell Adaptor Has a Dual Role in T-Cell Antigen Receptor Intracellular Signaling
Source: Front Immunol. 2018 Feb 2;9:115. doi: 10.3389/fimmu.2018.00115 (PMC5801411; doi:10.3389/fimmu.2018.00115)

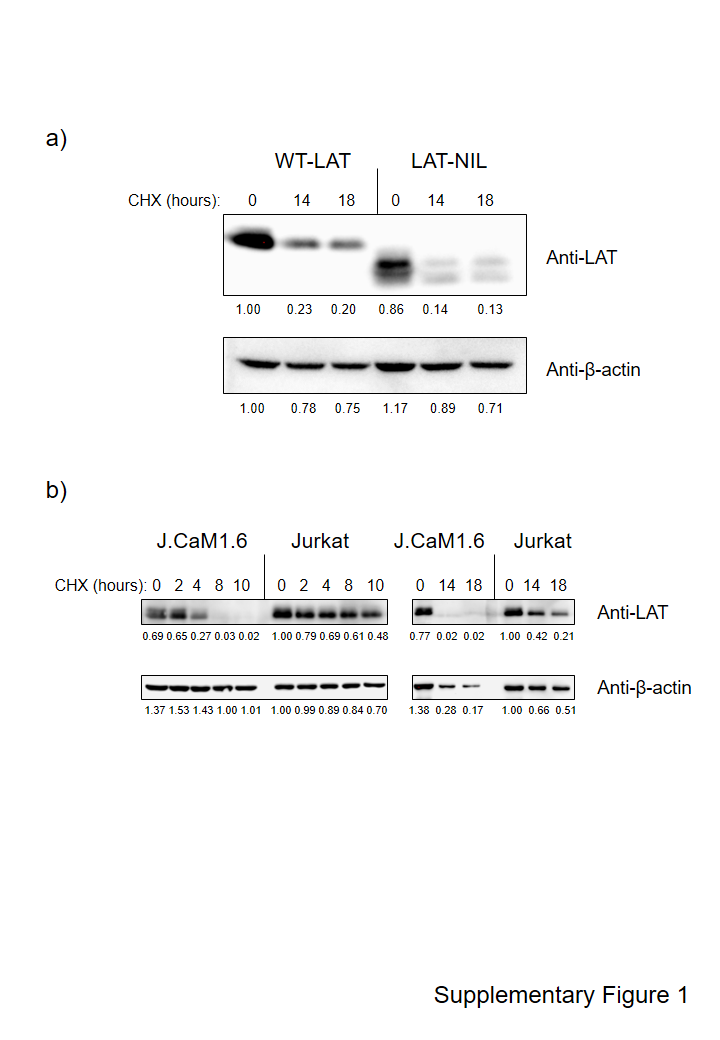

Supplement: Figure S1 — Stability of WT-linker for activation of T cell (LAT) and LAT-NIL mutant proteins. (A) J.CaM2 cells expressing WT-LAT or the LAT-NIL mutant were treated with cycloheximide (CHX) for indicated times in hours, and LAT and beta-actin protein levels were detected by Western blot. (B) Jurkat cells or the Lck-deficient cell line J.CaM1.6 were treated with cycloheximide (CHX) for indicated times in hours, and LAT (upper panels) and beta-actin (lower panels) protein levels were detected by Western blot. Images are representative of three independent experiments with similar results. Numbers below the blots indicate the densitometric quantification of the corresponding bands. [file image_1.tif]

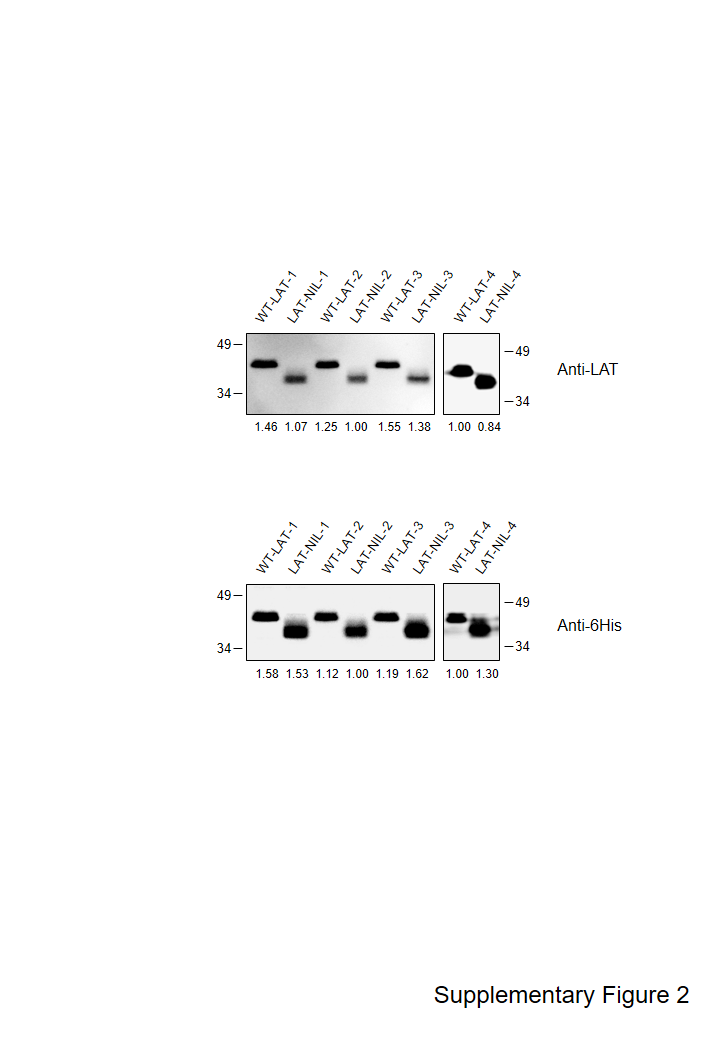

Supplement: Figure S2 — Expression of WT-linker for activation of T cell (LAT) and LAT-NIL mutant proteins. J.CaM2 cells were lentivirally transduced to express WT-LAT or the LAT-NIL mutant, and the expression of LAT was analyzed 1, 2, 3, and 4 weeks after lentiviral transduction by Western blotting with anti-LAT antibody or anti-6His antibody. Numbers below the blots indicate the densitometric quantification of the corresponding bands. Numbers below the blots indicate the densitometric quantification of the corresponding bands. [file image_2.tif]

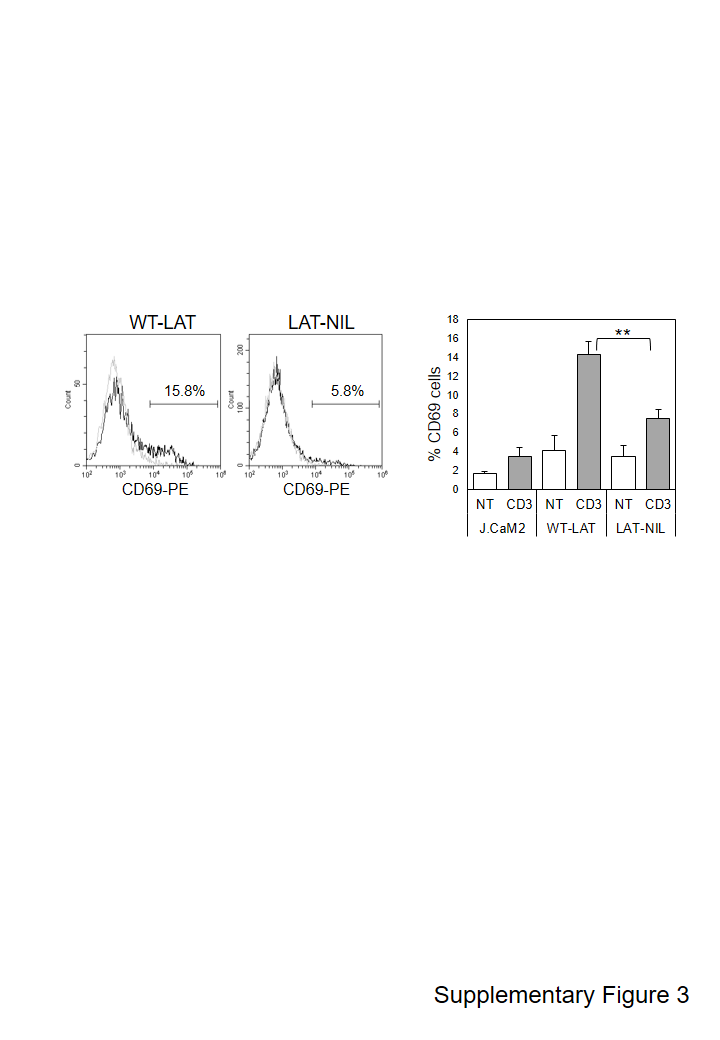

Supplement: Figure S3 — Negative impact of linker for activation of T cell (LAT)-NIL expression on activation-induced CD69 expression. Untransduced J.CaM2 cells or transduced with lentiviral vectors coding for WT-LAT or LAT-NIL were stimulated with immobilized anti-CD3 for 18 h at 37°C, and CD69 expression was analyzed by flow cytometry. Left and middle histograms show the result of a representative experiment showing CD69 expression (black line) in WT-LAT and LAT-NIL expressing cells. Gray lines indicate isotype-matched negative control antibody staining. Right panel shows average percentages of CD69+ cells in three independent experiments. Bars represent the standard error. Asterisks represent statistical significance. [file image_3.tif]
